# Network Pharmacology and Experimental Validation to Explore the Potential Mechanism of *Nigella sativa* for the Treatment of Breast Cancer

**DOI:** 10.3390/ph17050617

**Published:** 2024-05-10

**Authors:** Rawaba Arif, Shazia Anwer Bukhari, Ghulam Mustafa, Sibtain Ahmed, Mohammed Fahad Albeshr

**Affiliations:** 1 Department of Biochemistry, Government College University Faisalabad, Faisalabad 38000, Pakistan; 2 Scripps Institution of Oceanography, University of California San Diego, 9500 Gilman Drive, La Jolla, CA 92093, USA; 3 Department of Biochemistry, Bahauddin Zakariya University, Multan 60800, Pakistan; 4 Department of Zoology, College of Science, King Saud University, P.O. Box 2455, Riyadh 11451, Saudi Arabia

**Keywords:** breast cancer, molecular docking, phytochemicals, MDA-MB-231 cell line, DMBA

## Abstract

Breast cancer is a prevalent and potentially life-threatening disease that affects women worldwide. Natural products have gained attention as potential anticancer agents due to their fewer side effects, low toxicity, and cost effectiveness compared to traditional chemotherapy drugs. In the current study, the network pharmacology approach was used following a molecular docking study to evaluate the therapeutic potential of *N. sativa*-derived phytochemicals against breast cancer. Specifically, the study aimed to identify potential anticancer agents targeting key proteins implicated in breast cancer progression. Five proteins (i.e., EGFR, MAPK3, ESR1, MAPK1, and PTGS2) associated with breast cancer were selected as receptor proteins. Fourteen phytochemicals from *N. sativa* were prioritized based on drug-likeness (DL) and oral bioavailability (OB) parameters (with criteria set at DL > 0.18 and OB > 30%, respectively). Subsequent analysis of gene targets identified 283 overlapping genes primarily related to breast cancer pathogenesis. Ten hub genes were identified through topological analysis based on their significance in the KEGG pathway and GO annotations. Molecular docking revealed strong binding affinities between folic acid, betulinic acid, stigmasterol, and selected receptor proteins. These phytochemicals also demonstrated druggability potential. In vitro experiments in the MDA-MB-231 breast cancer cell line revealed that betulinic acid and stigmasterol significantly reduced cell viability after 24 h of treatment, confirming their anticancer activity. Furthermore, in vivo evaluation using a DMBA-induced rat model showed that betulinic acid and stigmasterol contributed to the significant recovery of cancer markers. This study aimed to explore the mechanisms underlying the anticancer potential of *N. sativa* phytochemicals against breast cancer, with the ultimate goal of identifying novel therapeutic candidates for future drug development. Overall, these results highlight betulinic acid and stigmasterol as promising candidates to develop novel anticancer agents against breast cancer. The comprehensive approach of this study, which integrates network pharmacology and molecular docking study and its experimental validation, strengthens the evidence supporting the therapeutic benefits of *N. sativa*-derived phytochemicals in breast cancer treatment, making them promising candidates for the development of novel anticancer agents against breast cancer.

## 1. Introduction

Among different cancers, breast cancer is a highly prevalent and life-threatening one, which affects women around the world and is responsible for significant rates of morbidity and mortality. According to an estimate, at some point in the lifetime of women, one in eight women will develop breast cancer [1]. Invasive breast cancer had approximately 297,790 new cases among US women, according to the American Cancer Society, as well as an estimated 55,720 cases of non-invasive breast cancer, in 2023 [2]. Triple-negative breast cancer (TNBC) is challenging, with a poor prognosis and limited treatment options, and is mainly managed with adjuvant chemotherapy. Treatment of TNBC requires the identification of additional receptor proteins and the development of corresponding drug ligands [3]. Patients with metastatic or advanced breast cancer face therapeutic limitations, including side effects, drug resistance, and a lack of selectivity, requiring the development of safer and more effective treatments.

Natural products have gained attention as potential anticancer agents due to their low toxicity, cost effectiveness, and fewer side effects compared to traditional chemotherapy drugs [4]. Studies have shown that natural products possess anticancer activities against breast cancer through a variety of mechanisms. For example, natural products can inhibit angiogenesis (i.e., a process in which tumors form new blood vessels to support their growth). Inhibition of angiogenesis can prevent tumor growth and impede metastasis [5]. Natural products can also inhibit cell migration and proliferation, which are key processes involved in tumor progression. By slowing or stopping cancer cell proliferation, natural products can prevent tumor growth and the spread of tumors [6]. Natural products can also regulate signaling pathways involved in cancer progression, such as Notch, PI3K/Akt/mTOR, NF-κB, MAPK/ERK, and NFAT-MDM2. By inhibiting or activating these signaling pathways, natural products can regulate gene expression, cell proliferation, and cell death [7]. Natural products can also regulate the epithelial-mesenchymal transition (EMT) process, which is an important step in cell invasion and metastasis of cancer. By inhibiting EMT, natural products can prevent the spread of cancer cells to other body parts [8].

The seeds of *Nigella sativa*, commonly known as black cumin, harbor a rich reservoir of phytochemicals renowned for their potent anticancer properties [9]. For millennia, the seeds of black cumin have held a revered status in traditional medicine for their therapeutic properties. The research findings of multiple studies underscore the ability of *N. sativa* extracts and its active constituents to induce apoptosis, inhibit cell proliferation and migration, and suppress tumor growth in various cancer cell lines and animal models. Furthermore, the plant exhibits notable analgesic and anti-inflammatory activities, suggesting its potential utility in managing pain and inflammatory conditions [10]. The diverse pharmacological repertoire of *N. sativa* positions it as a promising candidate for the development of novel therapeutic agents that target a spectrum of diseases, including cancer, pain, and inflammation.

Network pharmacology (NP), a relatively recent analytical approach, facilitates the comprehensive examination of drugs and diseases through multitarget and multipathway analysis. NP and molecular docking techniques synergistically contribute to elucidating the therapeutic potential of phytochemicals from *N. sativa* against breast cancer. They provide valuable insights into the complex interactions between phytochemicals and disease-related proteins, ultimately guiding the discovery and development of new anticancer agents derived from natural sources such as *N. sativa*. By integrating diverse databases, this method constructs a “drug active ingredient disease” model, allowing for the visualization of the underlying action mechanisms. Through this approach, researchers can gain insight into the intricate interplay between drugs and their targets, leading to comprehensive predictions about therapeutic targets and pathways [11]. The current investigation aimed to evaluate the therapeutic potential of phytochemicals derived from *N. sativa* for breast cancer treatment, utilizing an integrated strategy involving network pharmacology and molecular docking approaches followed by their in vitro and in vivo validation. Through these methods, our goal was to forecast the essential constituents, targets, and molecular pathways involved in the efficacy of *N. sativa* against breast cancer. Our findings reveal novel mechanistic insights into the therapeutic efficacy of *N. sativa* bioactive compounds against breast cancer, providing compelling evidence to support their translational potential in clinical settings.

## 2. Results

### 2.1. Network Pharmacology Study

#### 2.1.1. Active Constituents of *Nigella sativa*

A library of 283 phytochemicals was constructed. These phytochemicals were collected from the IMMPAT database. Based on pharmacokinetic parameters such as drug-likeness (DL > 0.18) and oral bioavailability (OB > 30%), only 14 phytocompounds were chosen for the network pharmacology study (Table S1).

#### 2.1.2. Target Prediction for *Nigella sativa*

The gene targets associated with *N. sativa* were predicted through the SwissTargetPrediction and STITCH databases, resulting in the prediction of 1415 targets for 14 phytochemicals. Subsequently, 32 distinct targets of drug-related genes were identified via post-elimination of duplicates through the alignment of UniprotKB protein IDs.

#### 2.1.3. Screening of Breast Cancer-Related Targets

**Figure 1:**
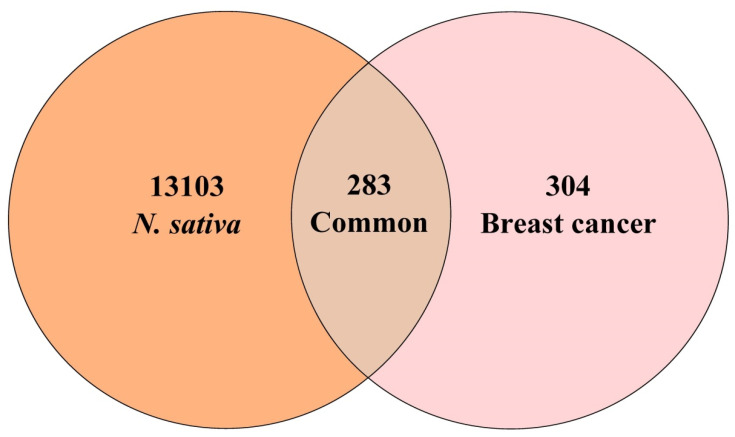
Venn diagram showing intersection of *N. sativa*-related targets and breast cancer-related predicted targets.

The GeneCard and DisGeNet databases were used, and 13,559 breast cancer-related gene targets were retrieved in total. After removing duplicates, a total of 13,386 unique genes were found. A comparison was made between *N. sativa*-related targets and predicted breast cancer targets to identify common and overlapping gene targets. Following this analysis, 283 total target genes were chosen through interactions of compound-related gene targets and breast cancer-related gene targets (Figure 1).

#### 2.1.4. Network Construction of Phytochemicals and Their Targets

The compound–target network was constructed for 14 active phytocompounds and 283 potential gene targets using Cytoscape software. This network, characterized by a dense interconnection of compounds and targets, was visually represented through a diagram (Figure S1), comprising 331 nodes and 1402 edges. The Network Analyzer plug-in was utilized to compute various network topological parameters. In particular, the network showed a density of 0.026, heterogeneity of 2.325, and centralization of 0.283, providing insight into interconnectedness, complexity of interactions within the network, and influence of specific nodes, respectively. In addition, the active phytochemicals were classified according to degree method, highlighting their interactions with numerous targets. In the study of network pharmacology, parameters such as network density, centralization, and heterogeneity offer quantitative measures to characterize the organization and functional properties of biological networks, elucidating the intricate relationships between genes, proteins, drugs, and other molecules.

#### 2.1.5. Protein–Protein Interactions

**Figure 2:**
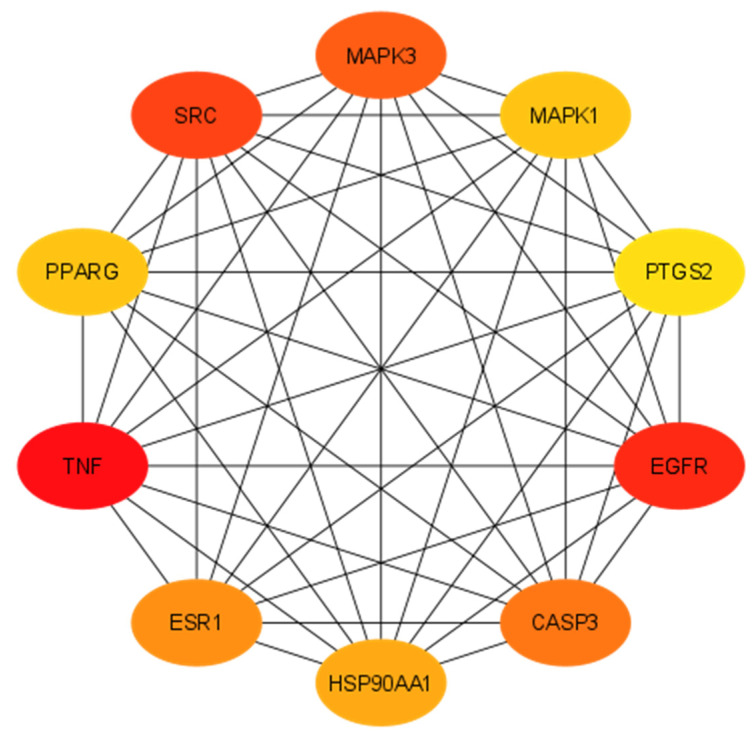
Top 10 hub genes as targets of *N. sativa* on breast cancer analyzed by Cytoscape.

The STRING 11.5, an online tool, was used to reveal protein–protein interactions (PPI). A total of 293 potential targets was taken into account, focusing on interactions with a score of 0.700, which is considered a high confidence score. *Homo sapiens* was used as the default organism (Figure S2). The resulting PPI network comprised 283 nodes and 3099 edges with 22 average node degrees. In a string PPI network, proteins produced by a single protein-coding gene locus are represented by nodes, protein–protein interactions are represented by edges, which means that proteins are jointly contributing to a shared function, and these might not physically bind to each other. The interaction types are elaborated in Table S2. Subsequently, the Network Analyzer tool within Cytoscape software was utilized to compute the network parameters. The network exhibited a density of 0.068, heterogeneity of 0.937, and centralization of 0.351. Furthermore, the CytoHubba plug-in was used to identify hub genes within the network, where the degree of the network signifies the number of interactions as a node (e.g., compound, gene, protein) engages with other nodes. Nodes with higher degrees are considered as hub nodes, exerting significant control or influence over the network’s functionality. The top targeted genes with their degrees are presented in Table 1 and illustrated in Figure 2.

#### 2.1.6. Functional Enrichment Analysis

**Figure 3:**
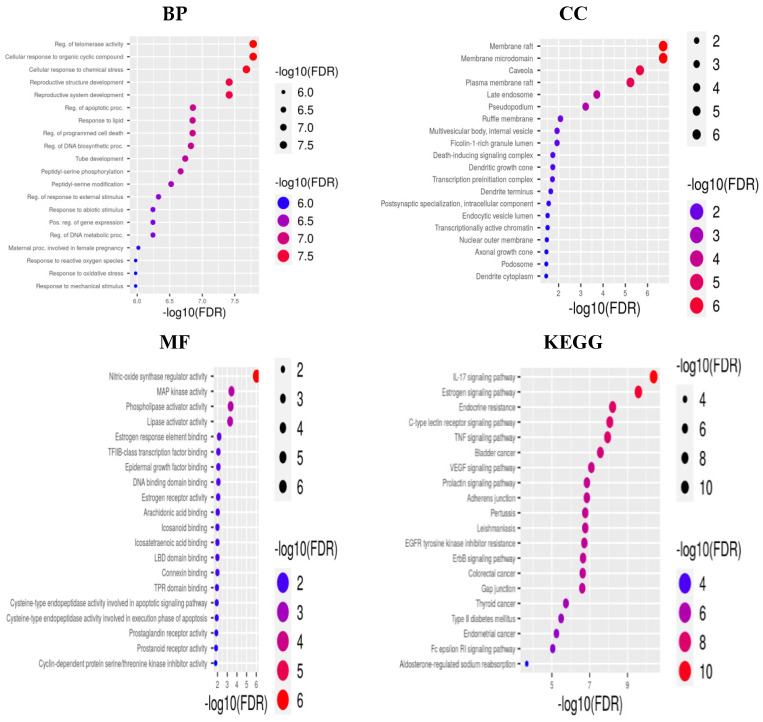
The bubble plot representation of the functional annotation and the enriched pathways in relation to breast cancer. (BP) GO with reference to biological processes, (CC) gene ontology with reference to cellular components, (MF) gene ontology with reference to molecular function, and KEGG pathway analysis.

Enrichment analysis was performed using the DAVID database to find functional annotation and to reveal pathway enrichment relevant to active phytochemicals for breast cancer treatment. The GO annotation exposed 128 biological processes (BP), 24 molecular functions (MF), and 22 cellular components (CC), along with 144 KEGG pathways. The top 20 terms identified in the GO and KEGG analyses primarily focused on cancer pathways (Figure 3). Biological processes encompass cellular responses to oxygen-containing components, cell proliferation, regulation of apoptosis, responses to various stimuli, including hormones, and hypoxia. The annotations of the cellular components included mitochondria, endoplasmic reticulum, receptor complexes, chromosomes, extracellular matrix, and membrane compartments. Molecular function terms are associated with various activities including ligand-activated transcription factor, phosphotransferases, nuclear receptor, ATP binding, and protein kinase. The KEGG pathway analysis highlighted the role and contribution of genes in cancer-related pathways such as prostate cancer, microRNAs, proteoglycans, endocrine resistance, and resistance to EGFR tyrosine kinase inhibitor. These pathways play a direct or indirect role in the onset of breast cancer.

**Figure 4:**
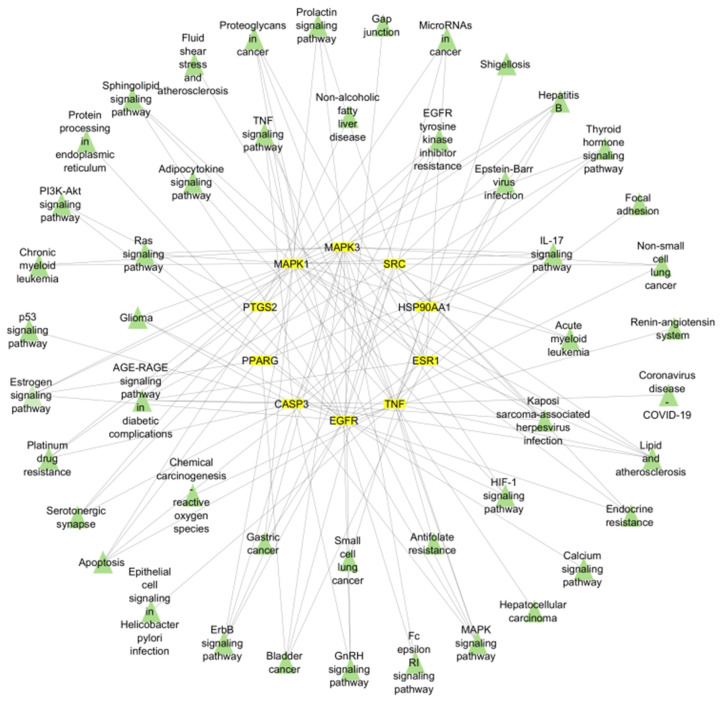
The network shows targets and their pathways. Network of 10 hub genes and top 20 pathways. The hub genes are highlighted in yellow, and the pathways are marked with green triangles.

The top 20 selected pathways were interpreted and visualized using the ShinyGO tool, generating a bar plot for easy comprehension. Furthermore, a target–pathway network was meticulously constructed using Cytoscape software to elucidate the intricate interrelation between targets and their associated signaling pathways (Figure 4).

#### 2.1.7. Construction of the Merged Compound–Target–Pathway Network

Cytoscape software was utilized to integrate the compound–target and target–pathway networks, allowing one to construct compound–target–pathway network. The network analyzer tool represented 14 active phytoconstituents, 10 hub genes, and 20 signaling pathways (Figure S3).

### 2.2. Ligand-Protein Interactions

The molecular docking technique was used to identify putative drug candidates for targeting specific genes identified through a network pharmacology study with the aim of treating breast cancer. Five key genes (EGFR, MAPK1, MAPK3, PTGS2, ESR1) were identified by network analysis, gene ontology (GO) and KEGG enrichment analyses. PyRx, a virtual screening software, was employed for molecular docking, while BIOVIA Discovery Studio was utilized for visualizing the 2D conformations of ligand and receptor protein affinities. The selection of the best drug candidates was made on the basis of good docking scores and RMSD values. PyRx provided information on the occupancy of the ligand within the active sites of the target molecules through docking scores. From a pool of 283 phytochemicals, the top two chemicals were individually chosen for each receptor protein based on their minimum docking scores and RMSD values (Table 2).

**Figure 5:**
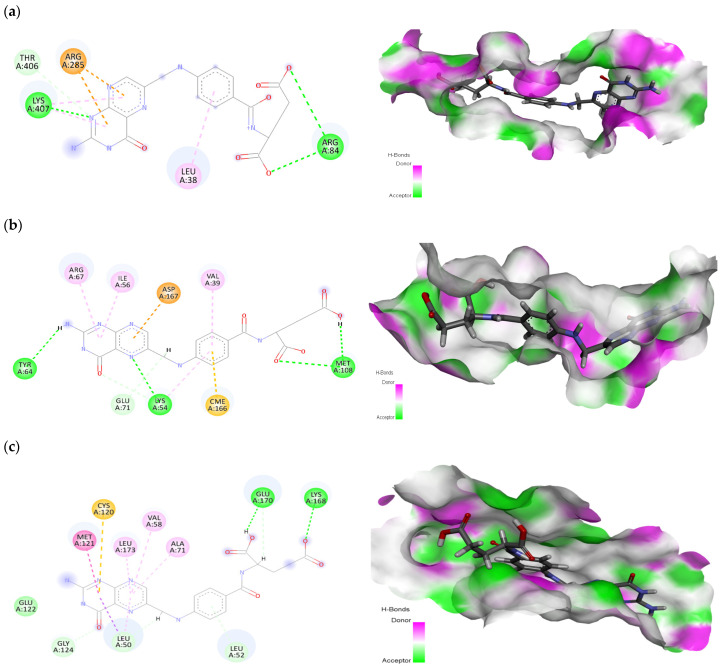

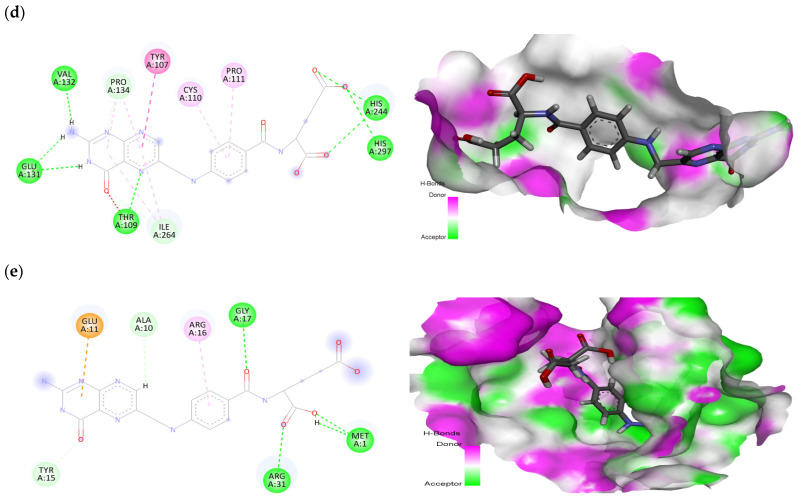
Interaction (left) and pocket fitting (right) of folic acid with (**a**) EGFR, (**b**) MAPK1, (**c**) MAPK3, (**d**) PTGS2, and (**e**) ESR1 as receptor proteins.

The phytochemical folic acid revealed interactions with all selected receptor proteins. Folic acid showed significant interactions with specific amino acids, namely Leu38, Arg84, Arg285, Thr406, and Lys407 within the EGFR protein binding pocket. These interactions resulted in a strong binding affinity, represented by docking score of −9.28 kcal/mol (Figure 5a). The amino acids Arg84 and Lys407 were involved in conventional hydrogen bonds, Leu38 was involved in π-alkyl interaction, the amino acids Arg285 and Thr406 were involved in π-cation and carbon hydrogen bond interactions, respectively. On the other hand, betulinic acid, the second-best phytochemical, showed interactions with Val6, Leu38, Phe263, and Tyr275 at the EGFR binding site, exhibiting a notable docking score of −7.27 kcal/mol (Figure S4). Within the MAPK1 protein, folic acid showed interactions with residues Val39, Lys54, Ile56, Tyr64, Arg67, Glu71, Met108, and Asp167, resulting in a docking score of −8.96 kcal/mol (Figure 5b). The residues Lys54, Tyr64, and Met108 made H-bonds, Val39, Ile56, and Arg67 made π-alkyl bonds, Glu71 was involved in the C-H bond, and Asp167 was involved in π-anion interaction with the folic acid ligand. In the same protein, stigmasterol interacted with Ile31, Val39, Lys54, Glu71, and Leu156 at the binding site, exhibiting a significant docking score of −6.33 kcal/mol (Figure S5). In the case of the MAPK3 protein, folic acid interacted with Leu50, Leu52, Val58, Ala71, Cys120, Met121, Glu122, Gly124, Lys168, Glu170 and Leu173, leading to a docking score of −8.98 kcal/mol (Figure 5c). Only two amino acids (i.e., Lys168 and Glu170) were involved in H-bonds, while three amino acids (i.e., Val58, Ala71 and Leu173) were involved in π-alkyl interactions. Amino acids Cys120 and Met121 were found to be involved in π-sulfur and amide-π stacked interactions. Glu122 was involved in van der Waals, while Leu50, Leu52, and Gly124 made a carbon H-bond with the ligand. Furthermore, stigmasterol, the second-best phytochemical, demonstrated interactions with Leu50, Val58, Ala71, Met118, Glu119, Cys120, Met121, and Leu173 at the MAPK3 binding site, with docking score of −8.57 kcal/mol (Figure S6).

Examining the PTGS2 protein, folic acid interacted with Tyr107, Thr109, Cys110, Pro111, Glu131, Val132, Pro134, His244, Ile264, and His297, resulting in a docking score of −8.14 kcal/mol (Figure 5d). In hydrogen bonding, the amino acids Thr109, Glu131, Val132, His244, and His297 were found to be involved. In the carbon H-bond, the residues Pro134 and Ile264 were involved. Cys110 and Pro111 made π-alkyl and Tyr107 made π-π stacked interactions with folic acid. Similarly, betulinic acid demonstrated interactions with Pro111, His244, Ile246, Tyr251, Ile264, Arg296, His297, and Val343 at the PTGS2 binding site, displaying a docking score of −6.95 kcal/mol (Figure S7). Within the ESR1 protein, folic acid was involved with Met1, Ala10, Glu11, Tyr15, Arg16, Gly17, and Arg31, leading to a docking score of −7.33 kcal/mol (Figure 5e). In H-bonds, the amino acids Met1, Gly17, and Arg31 were revealed to be involved. Ala10 and Tyr15 were involved in carbon H-bonds, while Glu11 and Arg16 were involved in π-anion and π-alkyl interactions, respectively. Similarly, betulinic acid interacted with Val8, Lys14, Val18, and Arg39 at the ESR1 binding site, exhibiting a docking score of −6.01 kcal/mol (Figure S8). Among the top-selected phytochemicals, folic acid, betulinic acid, and stigmasterol demonstrated strong and significant interactions with the selected receptor proteins.

### 2.3. Druggability Analyses

To be considered as potential drug candidates, the selected compounds must comply with a minimum of four Ro5 rules. Meeting these criteria suggests a high probability of oral bioavailability, making them suitable for further experimental studies including cell line and animal models [12]. The compounds analyzed violated only one of the five Ro5 rules (Table 3). According to previous research [13], a compound can be considered as a potential drug candidate if it violates one rule (out of five). To further evaluate the pharmacological potential of the selected phytochemicals from a medical perspective, the admetSAR tool was used. This tool provides additional information on ADMET-based attributes. The chosen phytochemicals confirmed their bioavailability as they met most of the druggability rules (Table S3). All selected phytocompounds were revealed to be noncarcinogens and to exhibit no Ames toxicity. On the basis of the overall drug profile, the phytocompounds selected in this study met the criteria of potential drug candidates in the drug discovery process.

### 2.4. In Vitro Evaluation of the Best Selected Phytochemicals in MDA-MB-231 Cells

**Figure 6:**
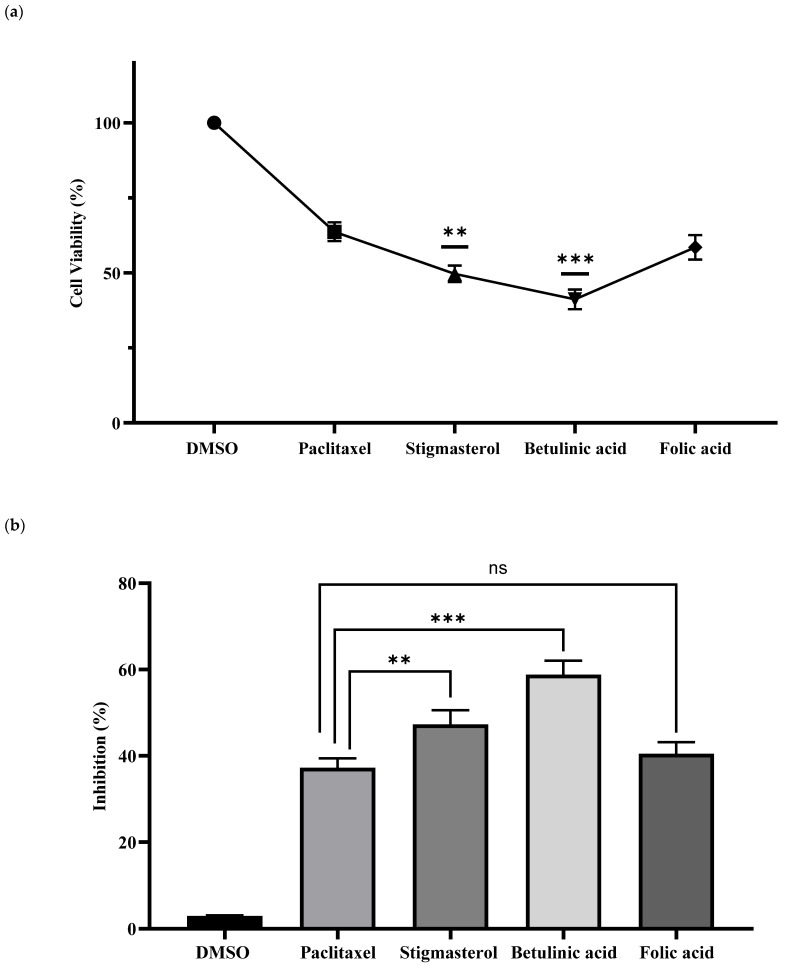
Cytotoxic activity of the best phytochemicals (i.e., folic acid, betulinic acid, and stigmasterol at concentrations of 200 µg/mL). (**a**) MTT assay for cytotoxicity analysis. The analysis was carried out in triplicate and the values are taken as mean ± standard error of the mean (SEM). (**b**) percentage of cell viability. The results are considered statistically very significant compared to the standard drug if the *p*-value was <0.01 and represented by ‘**’. When the *p*-value was found to be <0.001, it is considered highly significant compared to the standard drug and is represented by ‘***’. Values are shown as mean ± SEM for the representation of data. ns: nonsignificant.

After the molecular docking study, three phytochemicals (folic acid, stigmasterol, and betulinic acid) were selected for their in vitro evaluation in a breast cancer cell line. The cytotoxicity potential of selected phytochemicals was explored in MDA-MB-231 breast cancer cells using the MTT assay. Initially, various concentrations (i.e., 1.5625, 3.125, 6.25, 12.5, 25, 50, 100, and 200 µg/mL) of the three main compounds and the standard drug paclitaxel were used. The phytochemicals were applied to MDA-MB-231 cells for 24 h to assess their cytotoxicity potentials using the MTT test. To determine the inhibitory concentration (IC_50_) of the selected top compounds, the dose-response curves were generated. GraphPad Prism software (v8) was used to calculate IC_50_ values. Betulinic acid and stigmasterol showed the potential to significantly inhibit MDA-MB-231 cell growth with IC_50_ values of 14.52 µg/mL and 19.81 µg/mL, respectively. The cytotoxic potential of folic acid with an IC_50_ value of 32.39 µg/mL was found to be relatively lower against TNBC cells. The IC_50_ curves of selected compounds have been shown in Figures S9–S11 of the Supplementary Material. The percentage of cell viability and cytotoxicity potential of cells treated with DMSO, folic acid, betulinic acid, and stigmasterol are shown in Figure 6. The cytotoxicity of MDA-MB-231 cells was found to be significant and highly significant after treatment with stigmasterol (47.27%) and betulinic acid (58.05%), respectively, at a concentration of 200 µg/mL. Surprisingly, the phytochemical folic acid that showed strong binding interactions with all selected receptor proteins in the molecular docking study showed no significant inhibitory effect (41.44%) on MDA-MB-231 cell viability and exhibited almost the same inhibitory effect as paclitaxel.

### 2.5. In Vivo Evaluation

DMBA was used as a carcinogen in this study to induce breast cancer and two doses of 40 mg/kg DMBA dissolved in DMSO were administered to the rat groups except for the healthy group (control) and induction of the breast tumor took 12 weeks. After confirmation of breast cancer through the serum markers APF and CA125, rat group 3 was treated with the standard drug tamoxifen (35 mg/kg) dissolved in olive oil and administered orally daily for six weeks. Groups 4–7 were treated with low and high doses of stigmasterol and betulinic acid dissolved in olive oil and administered orally for six weeks.

#### 2.5.1. Effect of Stigmasterol and Betulinic Acid on Serum Parameters

**Figure 7:**
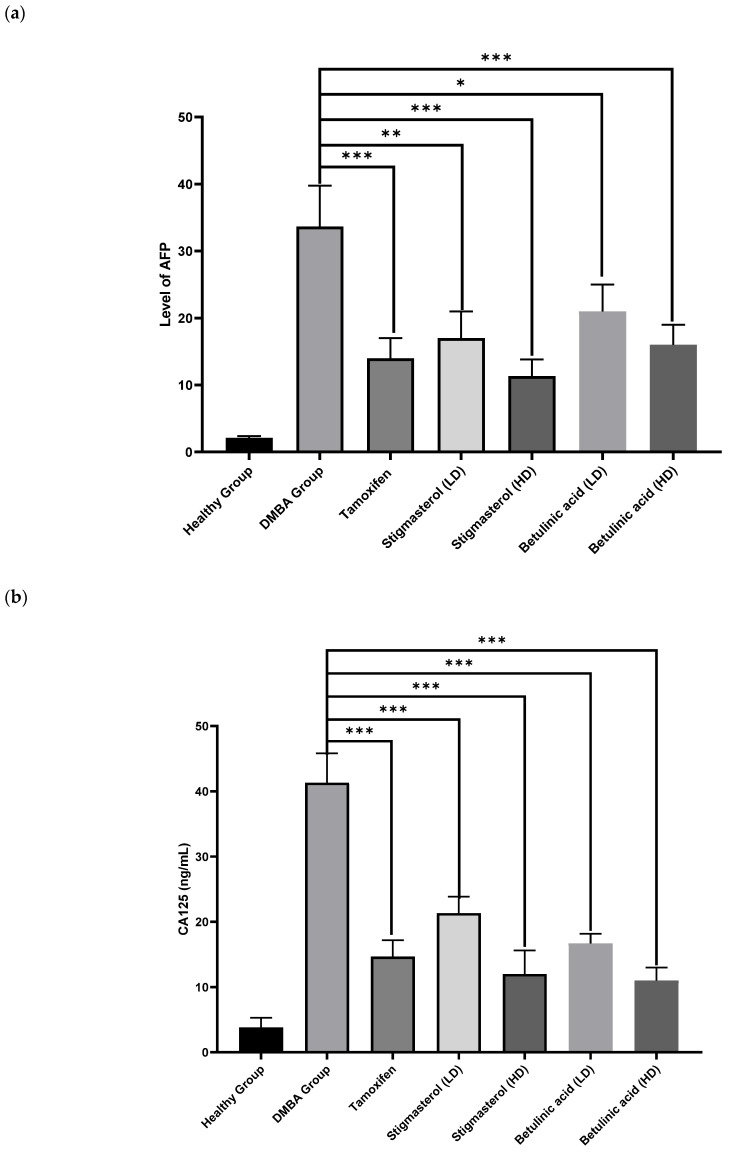
Effect of stigmasterol, betulinic acid, and standard drug on tumor markers. (**a**) The AFP values increased significantly in the DMBA group but recovered in the treatment groups. (**b**) The CA125 measurements. The results are considered statistically significant compared to the DMBA group if the *p*-value was <0.05, which is represented by ‘*’, if the *p*-value was <0.01 and represented by ‘**’. The standard drug tamoxifen (*p* < 0.001 ***), stigmasterol with low dose (LD) (*p* < 0.001 ***), stigmasterol high dose (HD) (*p* < 0.001 ***), betulinic acid low dose (LD) (*p* < 0.001 ***), and betulinic acid high dose (HD) (*p* < 0.001 ***) showed statistically highly significant results compared to the DMBA group.

Alpha-fetoprotein and cancer antigen 125 (CA125) are the standardized breast tumor markers. AFP and CA125 levels were found to be normal in the healthy group, but in the DMBA group, an elevation in the parameters of AFP and CA125 was observed (i.e., 33.6 ng/mL and 41.3 ng/mL, respectively). The high dose of betulinic acid showed a significant recovery of the AFP and CA125 parameters (i.e., 16.3 ng/mL and 11 ng/mL, respectively). The high dose of stigmasterol also showed significant recovery in the levels of both parameters (i.e., 11.3 ng/mL and 12 ng/mL, respectively) (Table 4, Figure 7). Compared to the standard drug tamoxifen, low and high doses of betulinic acid and stigmasterol showed significant results.

#### 2.5.2. Histology of Breast Tissue

**Figure 8:**
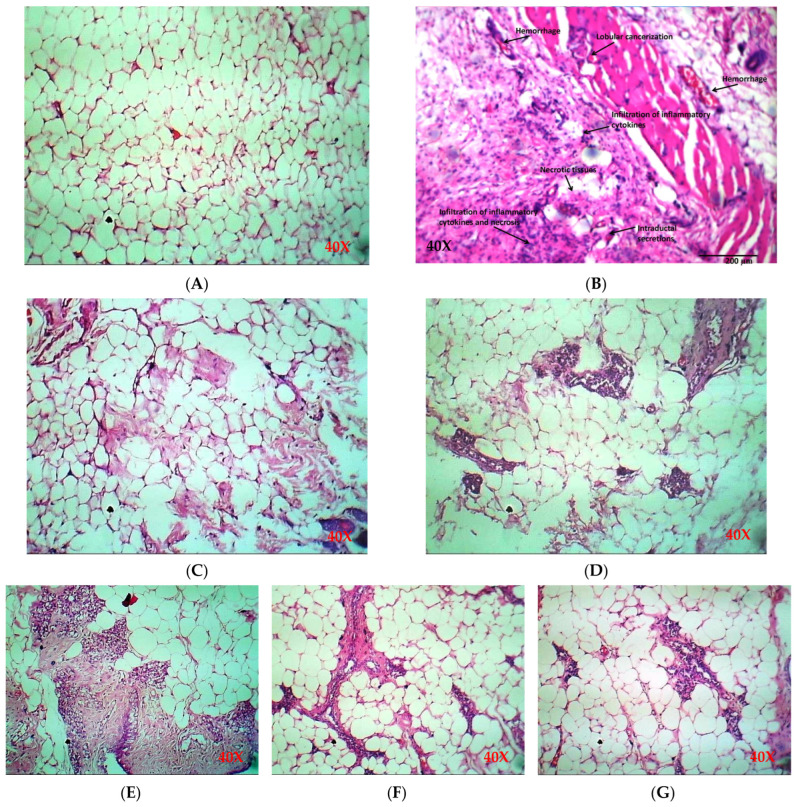
Histopathology of breast tissues. (**A**) mammary section of the healthy group (Group 1) showing no change in breast tissue, (**B**) mammary section of the DMBA group (Group 2), in which necrosis, hemorrhage, intraductal secretions, inflammation, and lobular cancerization were observed, (**C**) standard drug treatment (tamoxifen) (Group 3) showing significant improvement in breast tissue, (**D**) stigmasterol low dose group (Group 4) showing no inflammation, necrosis, or hemorrhage, (**E**) stigmasterol high-dose group (Group 5) showing improvement in breast tissue, (**F**) low-dose betulinic acid group (Group 6) representing no ductal secretions, inflammation, or necrosis, (**G**) high-dose betulinic acid group displaying significant improvement in breast tissue without evidence of cancer.

In the current study, a normal architecture of mammary tissue was shown by the mammary tissue section of the healthy group on histopathological examination. The DMBA group (cancer-induction group without treatment) showed intraductal secretions, necrosis, hemorrhage, infiltration in inflammatory cytokines, lobular cancerization, and mucin in the ductal lumen with highly granulated cytoplasm. Additionally, some ducts exposed discontinuity in the basement membrane with papillary outgrowth of cancerous cells and the variable degree of dedifferentiation suggesting adenocarcinoma. Meanwhile, the standard drug group and selected drug candidates stigmasterol and betulinic acid showed a significant improvement in breast tissues without inflammation, necrosis, and hemorrhage (Figure 8, Table 5). The histological characteristics of different groups of breast tissues have also been statistically analyzed and are given in Figure S12. The effects of low and high doses of stigmasterol and betulinic acid were compared with the DMBA group and were statistically significant for lobular cancerization, lobular carcinoma glands, ductal hyperplasia, acute inflammation, chronic inflammation, necrosis, and intraductal secretions.

## 3. Discussion

Addressing the challenges in preventing and treating breast cancer continues to be a global issue due to the lack of effective diagnostic biomarkers and therapeutic strategies. In recent years, there has been a growing interest in the use of herbal products derived from various plant sources as potential solutions for the prevention of cancer and the development of therapeutic agents [6]. Secondary metabolites of medicinal plant species have emerged as natural inducers of apoptosis signaling in various types of cancers [14]. Using network pharmacology techniques, researchers can explore complex interactions between ligands and target proteins, helping to identify and design novel therapeutic compounds [15].

This study focused on identifying key genes involved in the major signaling pathways associated with primary breast carcinoma for targeted treatment. Ten hub genes, including TNF, EGFR, SRC, MAPK3, CASP3, ESR1, HSP90AA1, MAPK1, PPARG, and PTGS2, were identified by topological analysis and protein–protein interactions (PPI). These genes were found to be prominently associated with the top 20 gene ontology annotations and KEGG pathways. Among them, EGFR, MAPK1, MAPK3, PTGS2, and ESR1 were specifically evaluated for breast cancer treatment. The study used network pharmacology to construct integrated compound–target–pathway networks, providing information on the complex relationships between drugs, targets, and signaling mechanisms in disease management. This approach enables the identification and selection of targets from a group of genes, enhancing the reliability of pinpointing genes involved in common cancer pathways [16].

Following the network construction between compounds, targets, and pathways, a comprehensive molecular docking analysis was conducted to investigate the interactions between phytochemicals as ligands and specific receptor proteins with their potential role in breast cancer. Folic acid was explored as the best phytocompound in the case of each receptor protein, while betulinic acid and stigmasterol were also revealed to have sufficiently strong binding interactions with receptor proteins. In the current study, the proteins EGFR, MAPK1, MAPK3, PTGS2, and ESR1 were used as target or receptor proteins in the docking analyses. Overexpression of the epidermal growth factor receptor (EGFR) has been implicated in numerous types of cancers, and inhibition of its activity has shown effectiveness in treating various cancers [17]. Similarly, MAPK1 and MAPK3 are components of the MAP kinase family and play crucial roles in cellular signaling. Extensive research studies have investigated both MAPK1 and MAPK3 as potential therapeutic targets for various types of cancers, including breast cancer [18,19]. Prostaglandin-endoperoxide synthase 2 (PTGS2), commonly known as cyclooxygenase-2 or COX-2, is an enzyme present in humans and is one of the two cyclooxygenases. PTGS2 expression is associated with inflammatory processes. PTGS2 has been explored as a potential target for cancer research studies [20]. The estrogen receptor alpha (ESR1) is a nuclear hormone receptor that mediates the effects of estrogen. It plays a critical role in promoting the growth of hormone receptor-positive breast cancers [21]. Endocrine therapies targeting ESR1 (e.g., tamoxifen, aromatase inhibitors) are standard treatments for hormone receptor-positive breast cancer [22].

In the current study, the phytochemicals betulinic acid and stigmasterol showed a significant inhibitory effect on the viability of MDA-MB-231 cells, which is consistent with other studies. Betulinic acid is a natural compound found in plants such as white birch. It has gained attention because of its potential therapeutic properties. It exhibits various biological activities, including anti-inflammatory, antiviral, antitumor, and antioxidant effects [23]. In a study, Cai et al. [24], explored the synergistic effect of betulinic acid with paclitaxel and found that arrest of the G2/M checkpoint of breast cancer cells and apoptosis were induced, and this synergistic effect exhibited little cytotoxicity effects on normal mammary epithelial cells. In another study, Zeng [25] explored the anti-metastasis activity of betulinic acid and revealed that the viability of three breast cancer cell lines (i.e., MDA-MB-231, MCF-7, and 4T1) was significantly decreased. They also determined that the administration of betulinic acid with a dose of 10 mg/kg/day inhibited tumor growth. The focal adhesion kinase (FAK)/MMP has been found to be critical for metastasis and cancer invasion [26]. In particular, the activation of MMP-2 and MMP-9 could enhance the potential for tumor cell metastasis in breast cancer. In another study [24], the cytotoxicity of betulinic acid against MDA-MB-231 was evaluated in various time intervals and revealed that the compound showed dose-dependent cytotoxicity effects on the cell line. The maximum value for the cell line was found to be 72 h and the IC_50_ of betulinic acid was determined to be 20.465 µM for MDA-MB-231. In another study, betulinic acid treatment significantly suppressed MDA-MB-231 cell proliferation, and the effect depends on the duration and concentration of betulinic acid. The increased concentration of betulinic acid has been found to be involved in the downregulation of an anti-apoptotic Bcl-2 gene [27].

Another phytochemical, stigmasterol, is a plant sterol found in soybeans, legumes, and vegetable oils. It is known for its potential health benefits and therapeutic properties. Research suggests that stigmasterol has anti-inflammatory, antioxidant, and anticancer effects [28]. In a study, Dube et al. [29] synthesized eight derivatives of stigmasterol and explored their antibreast cancer activity against the HCC70 cell line. They concluded that structural analogues of this phytochemical after modifications in functional groups can improve anticancer activity, and the compound and its derivatives have potential as lead anticancer drugs. Similarly, in a study [30], the antiproliferative effect of stigmasterol was explored and the IC_50_ at 48 h was found to be 90 µM in MDA-MB-231 cells. Omran et al., [31] studied the combined antitumor effects of stigmasterol and sorafenib against TNBC through MDA-MB-231 breast cancer cell lines and revealed that this combination could be a promising therapeutic option for treating breast cancer. In a study, Vundru et al. [32], have proposed that the treatment of MDA-MB-231 cells with stigmasterol could induce G0/G1 arrest in the cell line, which could be mediated by modulation in CDK-cyclin-CDKI protein levels.

Folic acid is a vital water-soluble nutrient essential for DNA synthesis, cell division, and growth. It is particularly important during periods of rapid cell growth, such as pregnancy and infancy. Folic acid is found in leafy greens, fruits, legumes, and fortified grains. Inadequate intake can cause anemia, fatigue, and cognitive impairment. It is crucial for red blood cell production and supports brain function. Deficiency has been linked to birth defects, such as neural tube defects [33]. Our study examined folic acid (folate) as a potential anticancer drug through a molecular docking study, but its inhibitory effect on the MDA-MB-231 cell line was found to be nonsignificant. In the literature, both positive and negative aspects of folic acid have been highlighted. Case-control studies demonstrated a statistically significant decrease in breast cancer risk with increased dietary folate intake. In a meta-analysis of studies by Ren et al. [34], published before April 2019, 39 studies on folate intake and 12 studies on plasma folate level were identified. Analysis revealed that higher folate intake was linked with a lower breast cancer risk compared to the highest and lowest categories. Similarly, Zeng et al. [35] performed a thorough analysis of case-control and cohort studies and found that higher folate intake, along with vitamin B6 and B2, may be associated with a lower risk of developing breast cancer.

In the current study, DMBA was used to induce breast cancer in female rats. The inhibitory or anti-breast-cancer effects of betulinic acid and stigmasterol were explored in animal models induced by DMBA by examining levels of cancer markers. These markers (e.g., alpha-fetoprotein (AFP) and cancer antigen 125 (CA125)) are molecules that are produced by cancer cells and could be used to diagnose breast cancer [36]. In this study, high doses of betulinic acid and stigmasterol treatment significantly recovered AFP and CA125 levels in DMBA-induced breast cancer rat models. The AFP biomarker belongs to aggressive malignant disease, and the fetal protein is used as a constituent member of panels of many combinations of biomarker assays to diagnose various cancers including breast cancer [37]. CA125 is a product of the MUC16 gene and is an important regulator of multiple pathways involved in the cell survival of breast cancer and ovarian cancer. In clinical routine, the use of CA125 to predict breast cancer has been widely incorporated [38].

The current study offers a novel perspective by focusing on the molecular interactions of betulinic acid and stigmasterol as potential agents against breast cancer. The positive results of our molecular docking, along with the satisfaction of ADMET and Lipinski’s rule criteria following the MDA-MB-231 breast cancer cell line study, provide further support for considering these phytochemicals in breast cancer treatment strategies. Among all compounds analyzed in this study, betulinic acid and stigmasterol exhibited strong interactions with five receptor proteins, suggesting their potential as a potent anticancer agent for the treatment of various types of breast cancer. More research and development efforts are warranted to explore their efficacy and safety profiles for breast cancer treatment.

## 4. Materials and Methods

### 4.1. Network Pharmacology Study

#### 4.1.1. Screening of Active Constituents of Nigella sativa

The bioactive substances found in *Nigella sativa* have been derived from scholarly sources and the openly accessible database IMPPAT [39]. All anticipated phytocompounds were virtually screened for drug-likeness (DL) and oral bioavailability (OB) properties [40]. Phytocompounds fulfilling the criteria of OB > 30% and DL > 0.18 were selected for further analysis. Among various pharmacokinetic parameters, OB is crucial in the case of ADME (i.e., absorption, distribution, metabolism, and excretion) criteria. High OB typically indicates favorable drug-likeness for active compounds. The compounds are classified as having high OB with an oral bioavailability of ≥30% [41]. Similarly, the DL index plays a crucial role in the rapid screening of active compounds, serving as a qualitative measure in drug discovery process to assess the druggability of a lead compound. Within DrugBank, 0.18 is the average value of the DL index, and compounds that possess a DL index of 0.18 or higher are considered to demonstrate high druggability [42].

#### 4.1.2. Therapeutic Targets of *Nigella sativa*

Potential gene targets linked to the chosen active compounds of *N. sativa* were identified and gathered using the online accessible tool SwissTargetPrediction [43] and the STITCH database [44], with *Homo sapiens* selected as the species. The UniProtKB database was used to cross-reference the protein identifiers of every protein to eliminate any redundancies [45].

#### 4.1.3. Screening of Targets Related to Breast Cancer

Human genes linked with the pathology of breast cancer were sourced from the GeneCard [46] and DisGeNET databases [47]. These target genes were compared to UniProtKB IDs to eliminate redundancies. The Jvenn plug-in was used to obtain a Venn diagram [48] to identify the shared genes from targets related to *N. sativa* and breast cancer.

#### 4.1.4. Network Construction of Phytochemicals and Targets

To generate the network of potential components of *N. sativa* and shared targets, Cytoscape 3.9.1 software [49] was utilized. The topological characteristics of the constructed network were explored using the Network Analyzer plug-in of Cytoscape.

#### 4.1.5. Construction of the Protein–Protein Interaction Network and Identification of Main Targets

Protein–protein interactions (PPI) within the network are essential for elucidating the underlying mechanisms and gene co-expression [50]. The STRING database was used to integrate overlapping genes, with a confidence score threshold of 0.7 applied to identify PPI. Cytoscape software (v3.9.1) was used to import and visualize the constructed network and topological analysis was performed using Network Analyzer tool. The hub genes were characterized to the greatest degree within the network using the CytoHubba plug-in.

#### 4.1.6. GO and KEGG Pathway Enrichment Analyses

Gene enrichment analyses for Gene Ontology (GO) annotation and the Kyoto Encyclopedia of Genes and Genomes (KEGG) pathway of hub genes were conducted using the DAVID database [51]. DAVID serves as a functional annotation resource to categorize sets of genes on the basis of biological process (BP), cellular component (CC), and molecular function (MF). Additionally, analysis of the KEGG pathway offers insights into high-degree genome mapping, elucidating genes with different molecular interactions and biological processes. A significant threshold of *p* < 0.05 was applied to identify highly enriched pathways (i.e., top 20) to build a pathway target network using Cytoscape 3.9.1 software [16]. The bubble plot visualizations for the GO and KEGG were generated using ShinyGO 0.77, an online available tool [52].

#### 4.1.7. Active Compound–Target–Pathway Network (C-T-P)

The C–T–P network was constructed using Cytoscape 3.9.1 by integrating compound–target and target–pathway networks. This integrated network facilitates understanding of the interconnections between each gene and its significant pathways, which play roles in specific cascades related to biological and cellular signaling. Using this network, we aim to identify the most relevant targets for active components relevant to breast carcinoma.

### 4.2. Ligand–Protein Docking Study

In this study, molecular docking was performed for phytocompounds sourced from *N. sativa* against five target proteins (i.e., EGFR, MAPK1, MAPK3, PTGS2, and ESR1), with the objective of investigating their potential for treating or suppressing breast cancer. PyRx software (v0.8) was used for molecular docking analysis to reveal the binding modes of ligand–protein interactions [13,53]. This approach complements the findings of NP and confirms the phytochemicals as lead compounds. The three-dimensional (3D) structures of the chosen receptor proteins were downloaded from the protein data bank (RCSB-PDB) in .pdb format [54] and the PubChem database was used to retrieve chemical structures of the active phytoconstituents in .sdf format [55]. Subsequently, the target proteins were prepared structurally by deleting already bound ligand(s) and removing water molecules and the addition of polar hydrogen atoms. Subsequently, the Visualizer tool of BIOVIA Discovery Studio (v21.1.0.20298) [56] was employed to visualize and generate 2D/3D representations of interactions and maps of selected phytocompounds with target proteins.

### 4.3. Assessment of Pharmacokinetic Parameters

The assessment of ADME profiling for the selected compounds involved the use of freely accessible online servers, specifically SwissADME [57]. These platforms are commonly employed to evaluate various ADME parameters. Understanding these parameters is crucial in drug designing as it helps to identify possible drug candidates [58].

### 4.4. In Vitro Evaluation

#### MDA-MB-231 Cell Culture and Cell Viability Determination

Human breast cancer cell line MDA-MB-231 was cultured in Dulbecco’s modified eagle medium (DMEM) for optimal growth and maintenance. Cell lines were obtained from the Department of Zoology, GC University Faisalabad. The medium was provided with 10% FBS (fetal bovine serum) and antibiotics (i.e., 100 μg/mL streptomycin, and 100 units/mL penicillin). The medium was then incubated at 37 °C with 5% CO_2_ in a humidified atmosphere. The best selected phytochemicals, mixed with DMSO with a final DMSO concentration of 0.05%, were used to treat MDA-MB-231 breast cancer cells. The MTT (3-[4,5-dimethylthiazol-2-yl]-2,5 diphenyl tetrazolium bromide) assay was used to determine cell viability [59]. DMSO and paclitaxel were used as a control and a standard drug, respectively. The best three phytochemicals (i.e., folic acid, betulinic acid, and stigmasterol) were used to treat MDA-MB-231 cells. Briefly, 96-well plates were used to seed and plant MDA-MB-231 cancer cells and the plates were incubated for 12 h. Various concentrations (i.e., 1.5625, 3.125, 6.25, 12.5, 25, 50, 100, and 200 µg/mL) of the best selected phytocompounds and the control drug (i.e., paclitaxel) were delivered to the MDA-MB-231 cancer cells. Cells were also treated with MTT reagent (50 µL/mL) and incubated for 4 h at 37 °C. Subsequently, DMSO (i.e., 150 μL) was added to dissolve formazan crystals. The microplate reader (Thermo Scientific) was then used to calculate the absorbance at 490 nm and the percentage of cell viability was calculated.

$$1\%=\frac{\left[A490\left(control\right)-A490(treated)\right]}{A490(control)}\times 100$$

### 4.5. In Vivo Evaluation

The Ethics Committee of the Government College University, Faisalabad reviewed and approved the animal study (approval number: GCUF/ERC/331). The breast cancer carcinogen 7,12-dimethylbenz(a) anthracene (DMBA) was purchased from Sigma-Aldrich (St. Louis, MO, USA). Tamoxifen (AstraZeneca, Cambridge, England) was used as a standard drug. The chloroform used to anesthetize the rats was obtained from Sigma-Aldrich. The cancer antigen (CA-125) enzyme-linked immunosorbent assay (ELISA) kit (Carlsbad, CA, USA) was used as a breast cancer marker. Fifty-six (56) female albino rats ranging from 175 to 200 mg were selected and kept in the animal house at room temperature in aluminum wire cages. The rats were divided into seven groups, with eight rats in each group. Throughout the experiment, the rats were free for water and food. The diet of the rats was composed of corn (36.7%), bone flour (14.5%), wheat (36.6%), fish flour (4.8%), crushed palm kernel (7.3%), sodium chloride (0.3%), and vitamin complex. The experiment was carried out in the Department of Pharmacy, Government College University Faisalabad during the winter season.

#### 4.5.1. Grouping of Animals

Rats were divided into seven groups and eight rats were selected for each group.

Group 1: Healthy group (only diet was given)

Group 2: DMBA group (diet + DMBA was administered)

Group 3: Standard drug group (diet + DMBA + tamoxifen was administered)

Group 4: Treatment group 1 (diet + DMBA + low-dose stigmasterol)

Group 5: Treatment group 2 (diet + DMBA + high-dose stigmasterol)

Group 6: Treatment group 3 (diet + DMBA + low-dose betulinic acid)

Group 7: Treatment group 4 (diet + DMBA + high-dose betulinic acid)

DMBA was used twice in the entire study to induce breast cancer in female rats. All rats excluding those in Group 1 were injected with DMBA (i.e., 40 mg/kg) dissolved in dimethyl sulfoxide (DMSO). The oral dose of tamoxifen (i.e., 35 mg/kg) dissolved in olive oil was administered to Group 3 as a standard drug after breast cancer induction for six weeks. Betulinic acid and stigmasterol were used as drug candidates for the treatment of breast cancer. After induction of breast cancer, both phytochemicals were administered orally dissolved in olive oil at two different doses (i.e., 50 mg/kg as low dose (LD) and 100 mg/kg as high dose (HD).

#### 4.5.2. Blood Collection

Chloroform was used as anesthesia and blood from all groups was obtained through a heart puncture. Blood was collected in two different blood tubes, one for serum analysis and the other for hematological studies.

#### 4.5.3. Determination of Serum Parameters

An enzyme-linked immunosorbent assay (ELISA) kit was used to estimate cancer antigen 125 (CA-125) [60] and alpha fetoprotein (AFP) [61] following manufacturer’s instructions.

#### 4.5.4. Histopathology of Breast Tissues

Breast tissues were isolated from each group and fixed in 10% formalin solution. Acetone and xylene were then used consecutively to process it for dehydration and clearing. In a 56 °C hot air oven, the tissues were impregnated with liquid paraffin for 30 min. The block was cut using a rotary microtome to pieces that were 5 μm thick. The pieces were then moved to a floating bath to remove the wax and finally to an egg albumin-coated slide. After 30 min in a hot air oven (56 °C), the slide was cleaned with xylene. After that, the slide was submerged for two minutes in 100%, 90%, 70%, and 50% alcohol. The slides were dipped in 1% ammonia solution, placed under running water for 10 minutes, and then dipped in hematoxylin once again. After dipping the slide into a 2% eosin solution, it was stained and then cleaned three times with 100% alcohol. After air drying, the piece was mounted using DPX mounting material and coated with glass slide [62].

### 4.6. Statistical Analysis

For statistical analysis, the Graph Pad Prism software (v8) was used. A one-way analysis of variance (ANOVA) was used to measure the significance of inhibition. Results with a *p*-value < 0.05 were considered statistically significant [63].

## 5. Conclusions

Breast cancer remains a challenging disease to treat despite advances in treatment options. In this study, network pharmacology was performed followed by molecular docking and druggability analyses to investigate interactions between *Nigella sativa* phytochemicals and five receptor proteins associated with breast cancer. Among the ligand molecules examined, folic acid, betulinic acid, and stigmasterol exhibited the most favorable binding patterns against all of the receptor proteins studied. Furthermore, a drug-likeness assessment and an ADMET analysis were conducted specifically on the top three selected phytochemicals, revealing their potential as drug candidates to treat breast cancer. The viability of MDA-MB-231 cells also confirmed the cytotoxic potential of betulinic acid and stigmasterol. The in vivo study revealed that betulinic acid and stigmasterol showed significant recovery in the levels of α-fetoprotein and cancer antigen 125 in DMBA-induced breast cancer rats. The study findings highlight the potential of betulinic acid and stigmasterol as promising candidates for further investigation and development as anticancer agents for breast cancer. Future research should focus on validating these results in diverse experimental models and, ultimately, in clinical settings, to advance the practical application of these phytochemicals in breast cancer therapy. Such efforts are crucial for realizing the therapeutic potential of *N. sativa* compounds in improving outcomes for patients with breast cancer.

## Figures and Tables

**Table 1:** The degrees of the hub genes computed using Cytoscape.

Sr. No.	Genes	Full Name	Degrees
1	TNF	Tumor Necrosis Factor	117
2	EGFR	Epidermal Growth Factor Receptor	92
3	SRC	Proto-Oncogene Tyrosine-Protein Kinase Src	89
4	MAPK3	Mitogen-Activated Protein Kinase 3	88
5	CASP3	Caspase 3	83
6	ESR1	Estrogen Receptor 1	80
7	HSP90AA1	Heat Shock Protein 90 Alpha Family Class A Member 1	79
8	MAPK1	Mitogen-Activated Protein Kinase 1	76
9	PPARG	Peroxisome Proliferator-Activated Receptor Gamma	76
10	PTGS2	Prostaglandin-Endoperoxide Synthase 2	72

**Table 2:** Binding interactions of the top two phytochemicals with each receptor protein.

Sr. No.	Ligand	Receptor	Docking Score (kcal/mol)	Interacting Residues
1	Folic acid	EGFR	−9.28	Leu38, Arg84, Arg285, Thr406, Lys407
2	Betulinic acid		−7.27	Val6, Leu38, Phe263, Tyr275
3	Folic acid	MAPK1	−8.96	Val39, Lys54, Ile56, Tyr64, Arg67, Glu71, Met108, Asp167
4	Stigmasterol		−6.33	Ile31, Val39, Lys54, Glu71, Leu156
5	Folic acid	MAPK3	−8.98	Leu50, Leu52, Val58, Ala71, Cys120, Met121, Glu122, Gly124, Lys168, Glu170, Leu173
6	Stigmasterol		−8.57	Leu50, Val58, Ala71, Met118, Glu119, Cys120, Met121, Leu173
7	Folic acid	PTGS2	−8.14	Tyr107, Thr109, Cys110, Pro111, Glu131, Val132, Pro134, His244, Ile264, His297
8	Betulinic acid		−6.95	Pro111, His244, Ile246, Tyr251, Ile264, Arg296, His297, Val343
9	Folic acid	ESR1	−7.33	Met1, Ala10, Glu11, Tyr15, Arg16, Gly17, Arg31
10	Betulinic acid		−6.01	Val8, Lys14, Val18, Arg39

**Table 3:** Molecular properties of the best selected phytocompounds.

Ligands	Molecular Properties						Violations
	MW	HBD	HBA	Nrotb	LogP	*A*	
Stigmasterol	412.69	1	1	5	5.08	132.75	1
Folic acid	441.4	6	3	10	−0.25	111.92	1
Betulinic acid	456.7	2	9	2	3.83	136.91	1

MW: Molecular weight, HBD: H-bond donor, HBA: H-bond acceptor, Nrotb: No. of rotatable bonds, LogP: Logarithm of octanol/H_2_O coefficient.

**Table 4:** Effects of stigmasterol and betulinic acid on serum parameters.

Parameter	Groups						
	Healthy	DMBA	Standard Drug	Stigmasterol (LD)	Stigmasterol (HD)	Betulinic Acid (LD)	Betulinic Acid (HD)
AFP (ng/mL)	2.1 ± 0.25	33.6 ± 6.1	14 ± 3 ***	17 ± 4 **	11.3 ± 2.5 ***	21 ± 4 *	16 ± 3 ***
CA125 (ng/mL)	3.83 ± 1.45	41.3 ± 4.5	14.6 ± 2.5 ***	21.3 ± 2.5 ***	12 ± 3.6 ***	16.6 ± 1.5 ***	11 ± 2 ***

(*) significant (*p* < 0.05), (**) very significant (*p* < 0.01), and (***) highly significant (*p* < 0.001). Stars represent comparison of treatment groups with the standard drug, while all treatment groups showed highly significant results with the DMBA group discussed in the figures as well.

**Table 5:** Histological characteristics of breast tissues.

Breast Project								
Specimen ID	Lobular Cancerization	Lobular Carcinoma Glands	Ductal Hyperplasia	Acute Inflammation	Chronic Inflammation	Necrosis	Hemorrhage	Intraductal Secretions
G1	Intact	Intact	Intact	Not Seen	Not Seen	Not Seen	Not Seen	Seen
G2	Lobular cancerization	Invasive Lobular carcinoma +	Ductal hyperplasia ++	+++	+++	Fat necrosis +++	+++	+++
G3	Intact	Intact	Intact	Not Seen	Not Seen	Not Seen	Not Seen	Seen
G4	Slightly distorted	Intact	Intact	Not Seen	Mild Lymphocyte	Not Seen	Not Seen	Seen
G5	Slightly distorted	Intact	Intact	Not Seen	Mild Lymphocyte	Not Seen	Not Seen	Seen
G6	Intact	Intact	Intact	Not Seen	Not Seen	Not Seen	Not Seen	Seen
G7	Intact	Intact	Intact	Not Seen	Not Seen	Not Seen	Not Seen	Seen

KEY: Score 0: Not seen, score +: Mild, score ++: Moderate, score +++: Severe. G1: Healthy group (only diet was given), G2: DMBA group (diet + DMBA was administered), G3: Standard drug group (diet + DMBA+ tamoxifen was administered), G4: Treatment group 1 (diet + DMBA+ lose dose stigmasterol), G5: Treatment group 2 (diet + DMBA+ high dose stigmasterol), G6: Treatment group 3 (diet + DMBA+ low dose betulinic acid), G7: Treatment group 4 (diet + DMBA + high dose betulinic acid).

## Data Availability

Data is contained within the article and Supplementary Material.

## Supplementary Materials

The following supporting information can be downloaded at: https://www.mdpi.com/article/10.3390/ph17050617/s1, Figure S1. Compound–target network of active phytochemicals and their targets. Figure S2. PPIs showing the interactions of 283 target genes, colored nodes represent query protein; white nodes represent second shell of interactors; empty nodes represent protein with unknown 3D structure; filled nodes represent protein with known or predicted 3D structure. Figure S3. The network of the compound-target pathway (yellow: common target genes; light purple: compounds; green: pathways). Figure S4. Binding interaction (a) and pattern (b) of betulinic acid with the EGFR as a receptor. Figure S5. Binding interaction (a) and pattern (b) of stigmasterol with the MAPK1 as a receptor. Figure S6. Interaction (a) and binding pattern (b) of stigmasterol with the MAPK3 as a receptor. Figure S7. Interaction (a) and binding pattern (b) of betulinic acid with the PTGS2 as a receptor. Figure S8. Interaction (a) and binding pattern (b) of betulinic acid with the ESR1 as a receptor. Figure S9. Folic acid IC_50_ curve. Figure S10. Betulinic acid IC_50_ curve. Figure S11. Stigmasterol IC_50_ curve. Figure S12. Histological features of different groups of breast tissues with statistical significance. Table S1. The characteristics of the best selected phytocompounds. Table S2. Indication of each color interaction in the PPI network of STRING. Table S3. Drug-like parameters or ADMET profiling of the best selected phytochemicals.
