# Supplementary material for: Network Pharmacology and Experimental Validation to Explore the Potential Mechanism of Nigella sativa for the Treatment of Breast Cancer
Source: Pharmaceuticals (Basel). 2024 May 10;17(5):617. doi: 10.3390/ph17050617 (PMC11124279; doi:10.3390/ph17050617)
Supplement: Supplementary file 1 [file pharmaceuticals-17-00617-s001.zip › pharmaceuticals-2978847-supplementary.pdf]

Network Pharmacology and Experimental Validation to Explore the Potential Mechanism of *Nigella sativa* for the Treatment of Breast Cancer

Rawaba Arif[1], Shazia Anwer Bukhari[1]*, Ghulam Mustafa[1], Sibtain Ahmed[2, 3] and Mohammed Fahad Albeshr[4]

[1]Department of Biochemistry, Government College University Faisalabad, Faisalabad-38000, Pakistan

[2]Scripps Institution of Oceanography, University of California San Diego, 9500 Gilman Drive, La Jolla, CA 92093, USA

[3]Department of Biochemistry, Bahauddin Zakariya University, Multan-60800, Pakistan

[4]Department of Zoology, College of Science, King Saud University, PO Box 2455, Riyadh,11451, Saudi Arabia

*Correspondence: shaziabukhari@gcuf.edu.pk

Supplementary file

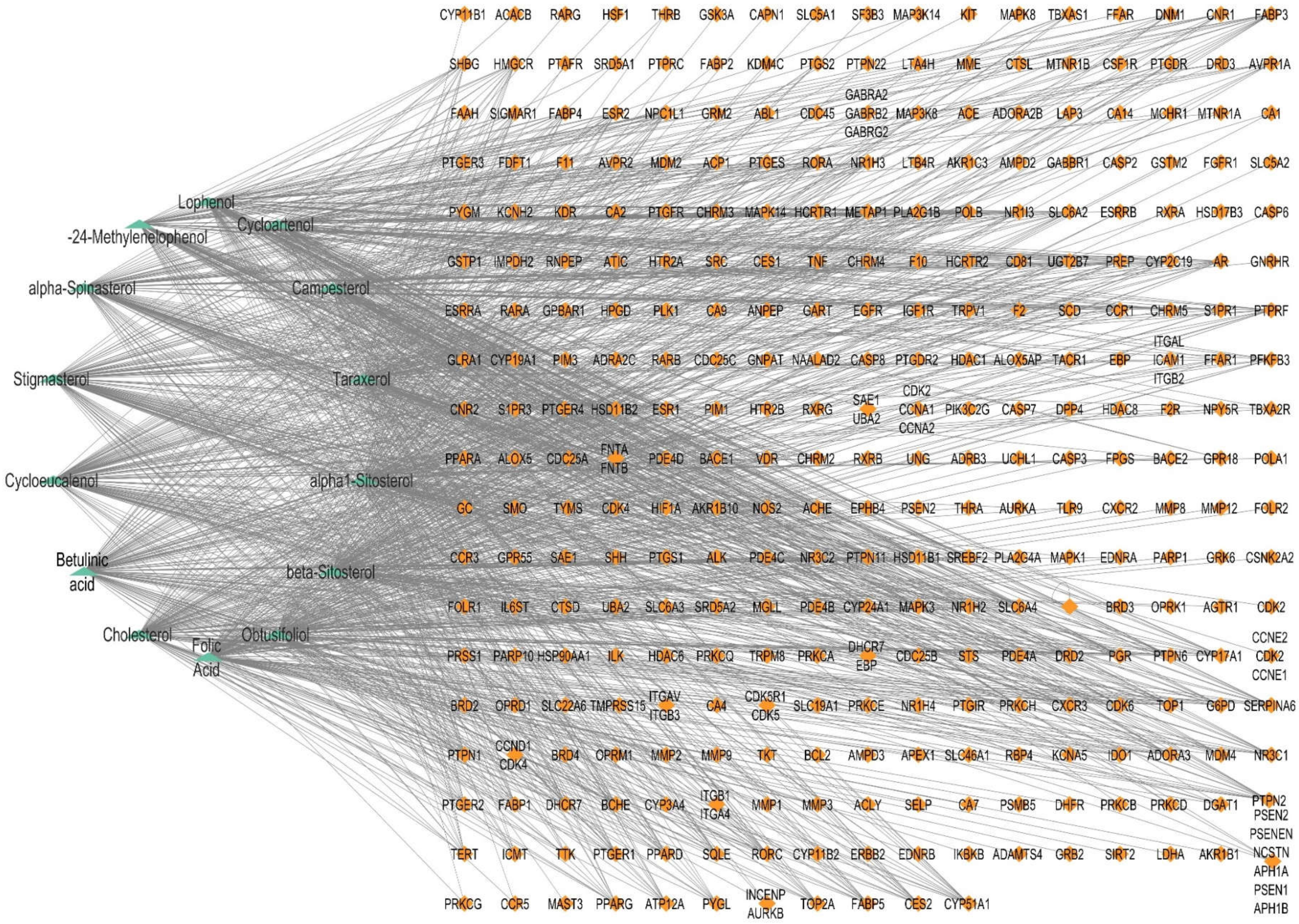

Figure S1. Compound–target network of active phytochemicals and their targets.

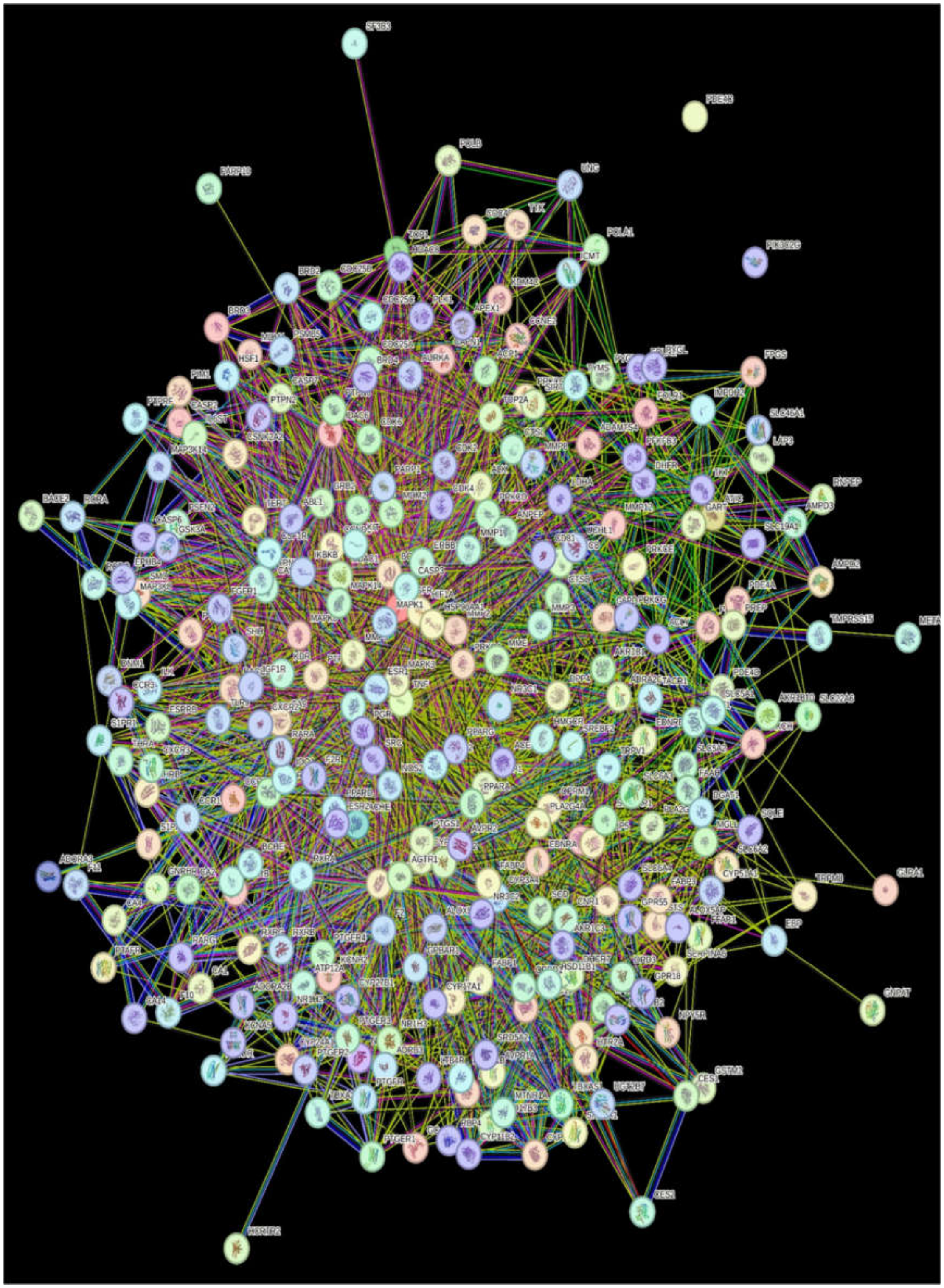

Figure S2. PPIs showing the interactions of 283 target genes, colored nodes represent query protein; white nodes represent second shell of interactors; empty nodes represent protein with unknown 3D structure; filled nodes represent protein with known or predicted 3D structure.

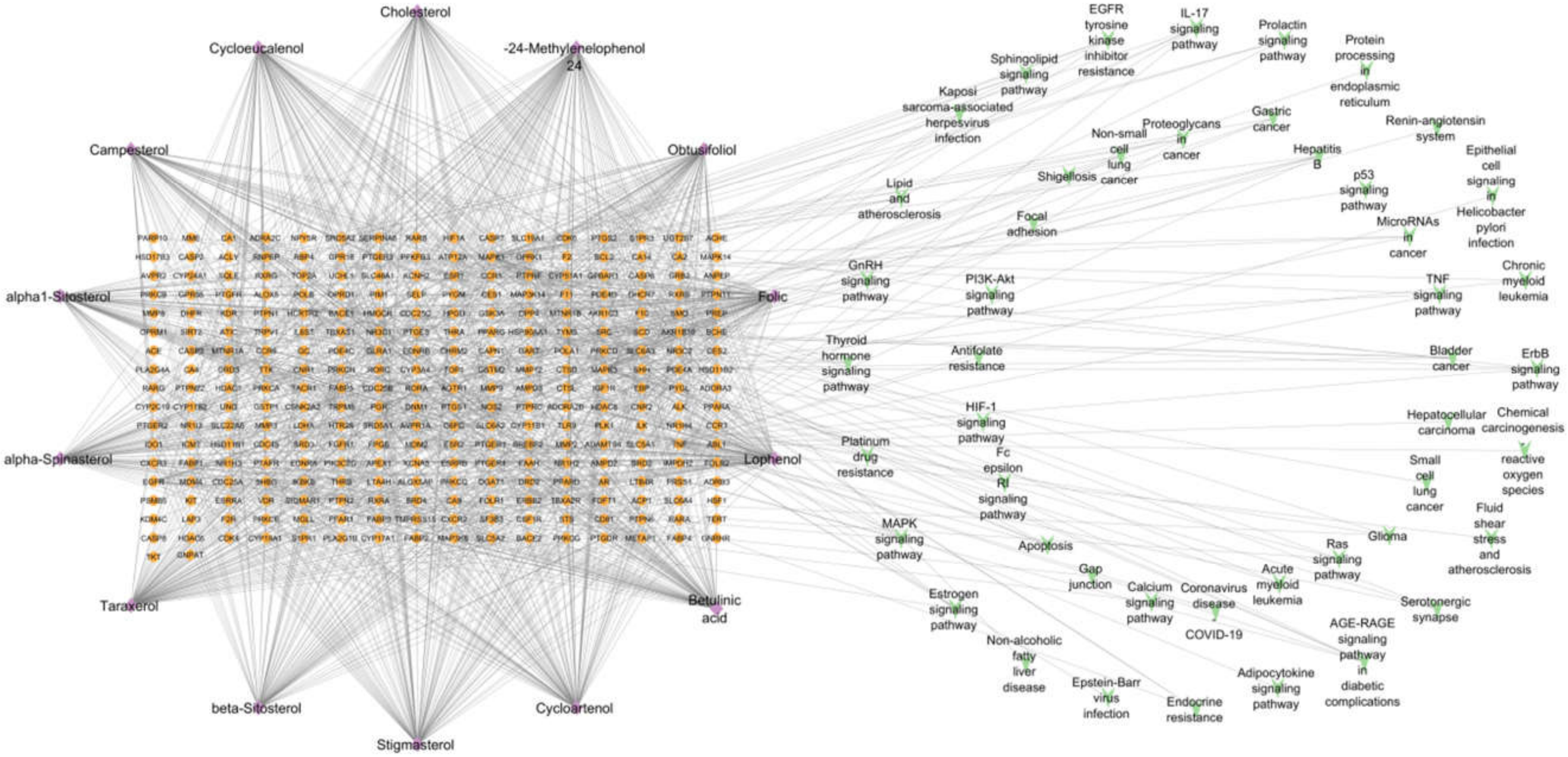

Figure S3. The network of the compound-target pathway (yellow: common target genes; light purple: compounds; green: pathways).

(a) (b)

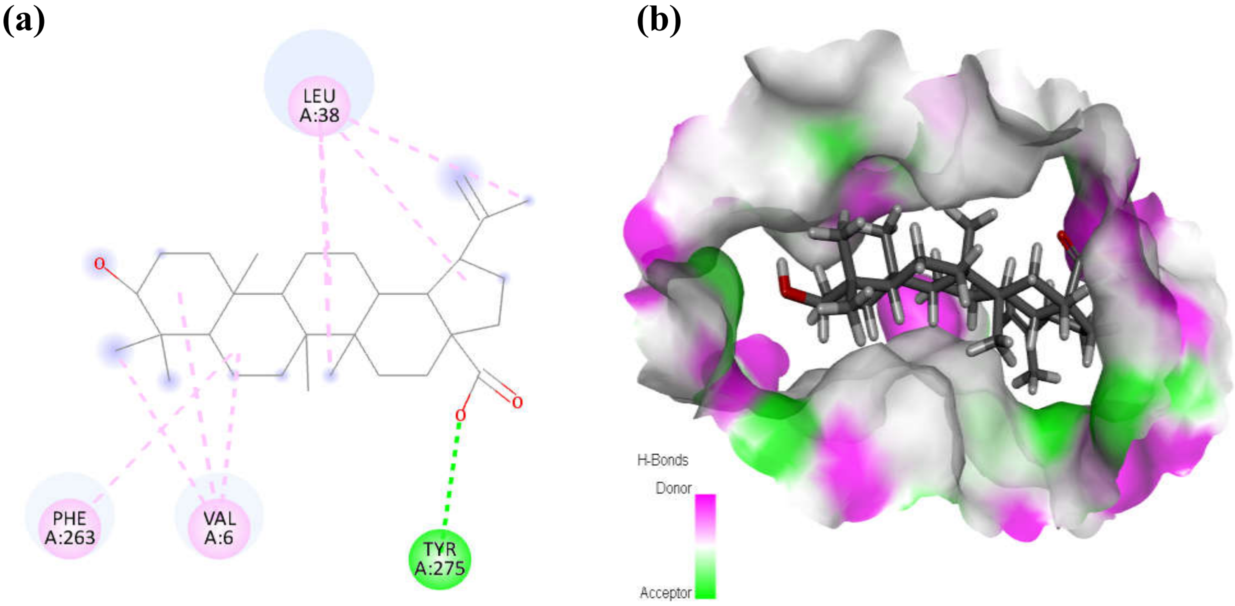

Figure S4. Binding interaction (a) and pattern (b) of betulinic acid with the EGFR as a receptor.

(a) (b)

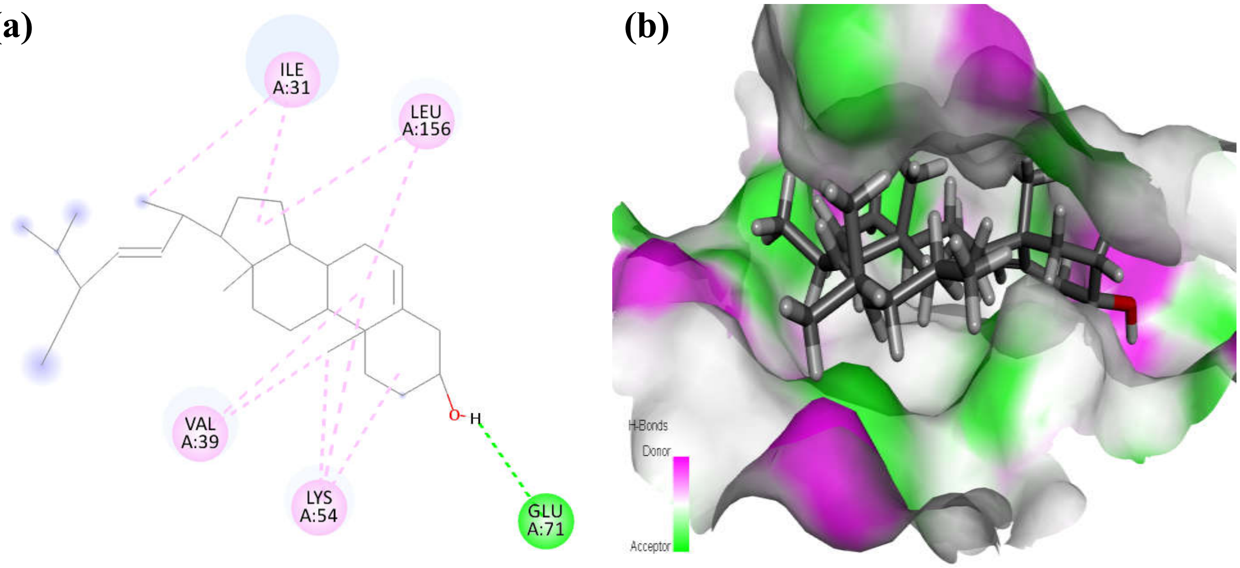

Figure S5. Binding interaction (a) and pattern (b) of stigmasterol with the MAPK1 as a receptor.

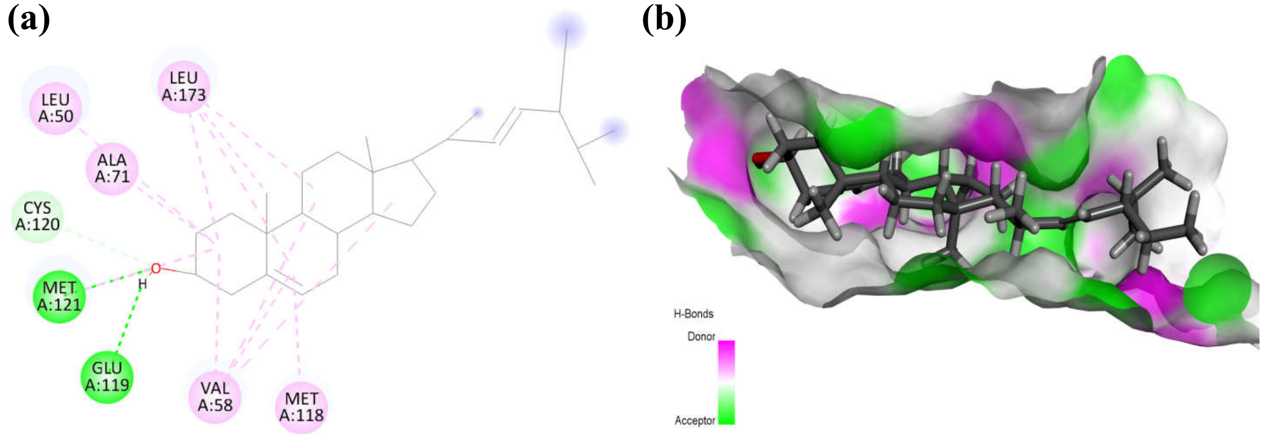

Figure S6. Interaction (a) and binding pattern (b) of stigmasterol with the MAPK3 as a receptor.

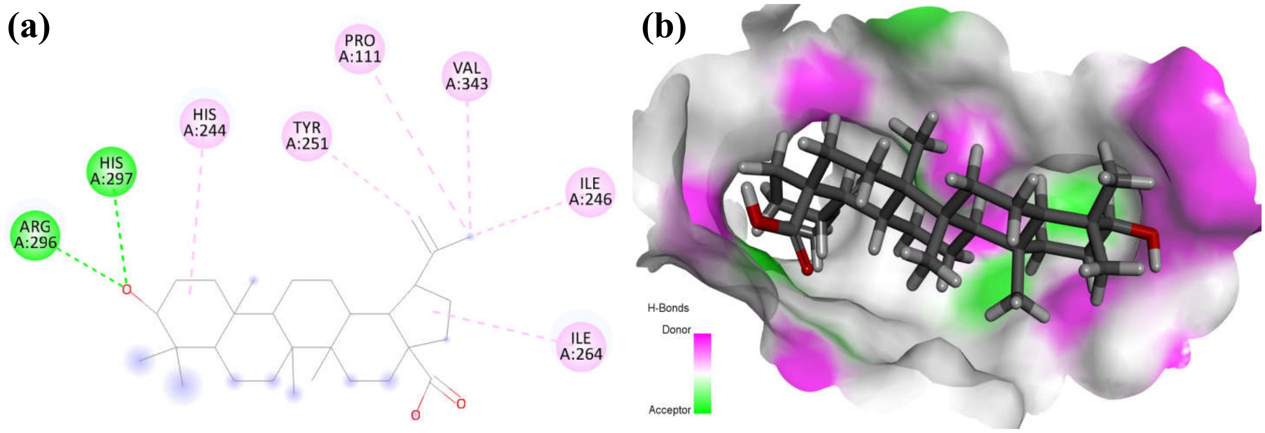

Figure S7. Interaction (a) and binding pattern (b) of betulinic acid with the PTGS2 as a receptor.

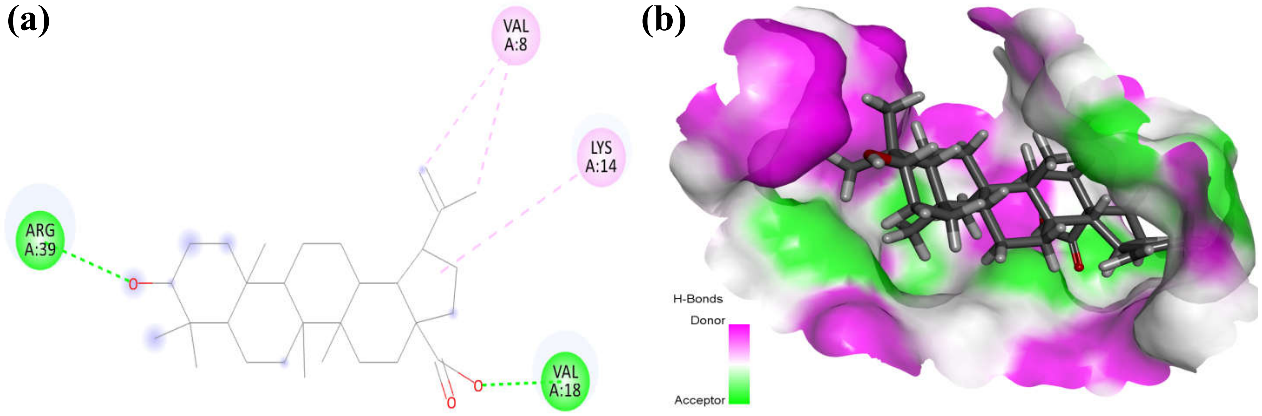

Figure S8. Interaction (a) and binding pattern (b) of betulinic acid with the ESR1 as a receptor.

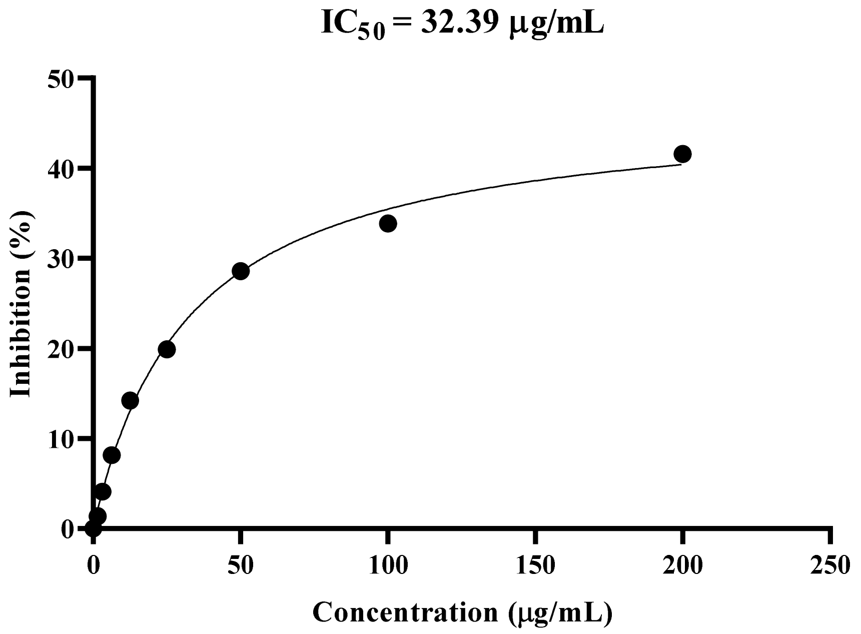

Figure S9. Folic acid IC_{50} curve

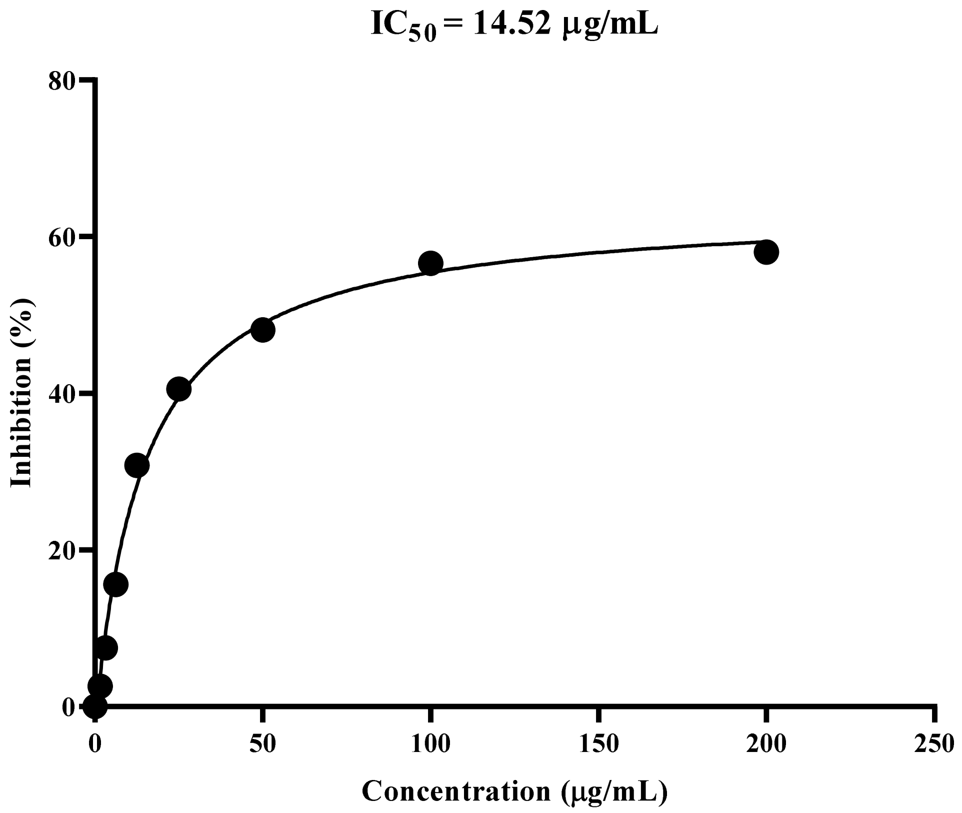

Figure S10. Betulinic acid IC_{50} curve.

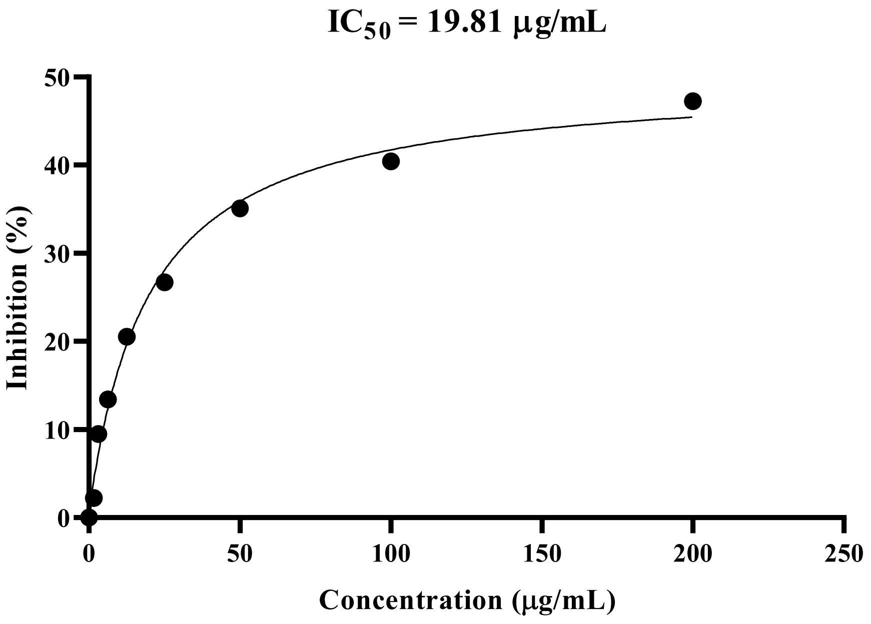

Figure S11. Stigmasterol IC_{50} curve.

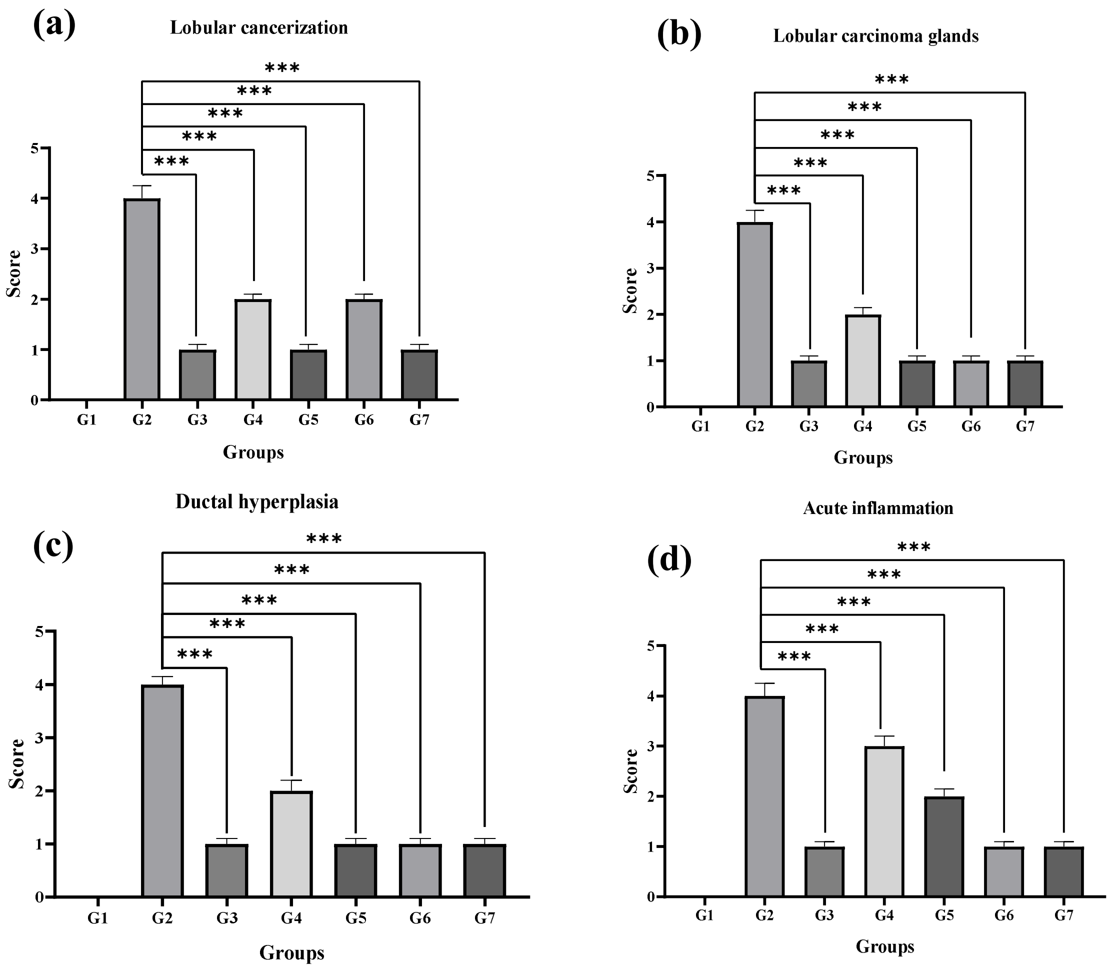

(a)
Lobular cancerization
***
***
***
***
***
Score
0
1
2
3
4
5
G1
G2
G3
G4
G5
G6
G7
Groups
(b)
Lobular carcinoma glands
***
***
***
***
***
Score
0
1
2
3
4
5
G1
G2
G3
G4
G5
G6
G7
Groups
Ductal hyperplasia
(c)
***
***
***
***
***
Score
0
1
2
3
4
5
G1
G2
G3
G4
G5
G6
G7
Groups
Acute inflammation
(d)
***
***
***
***
***
Score
0
1
2
3
4
5
G1
G2
G3
G4
G5
G6
G7
Groups

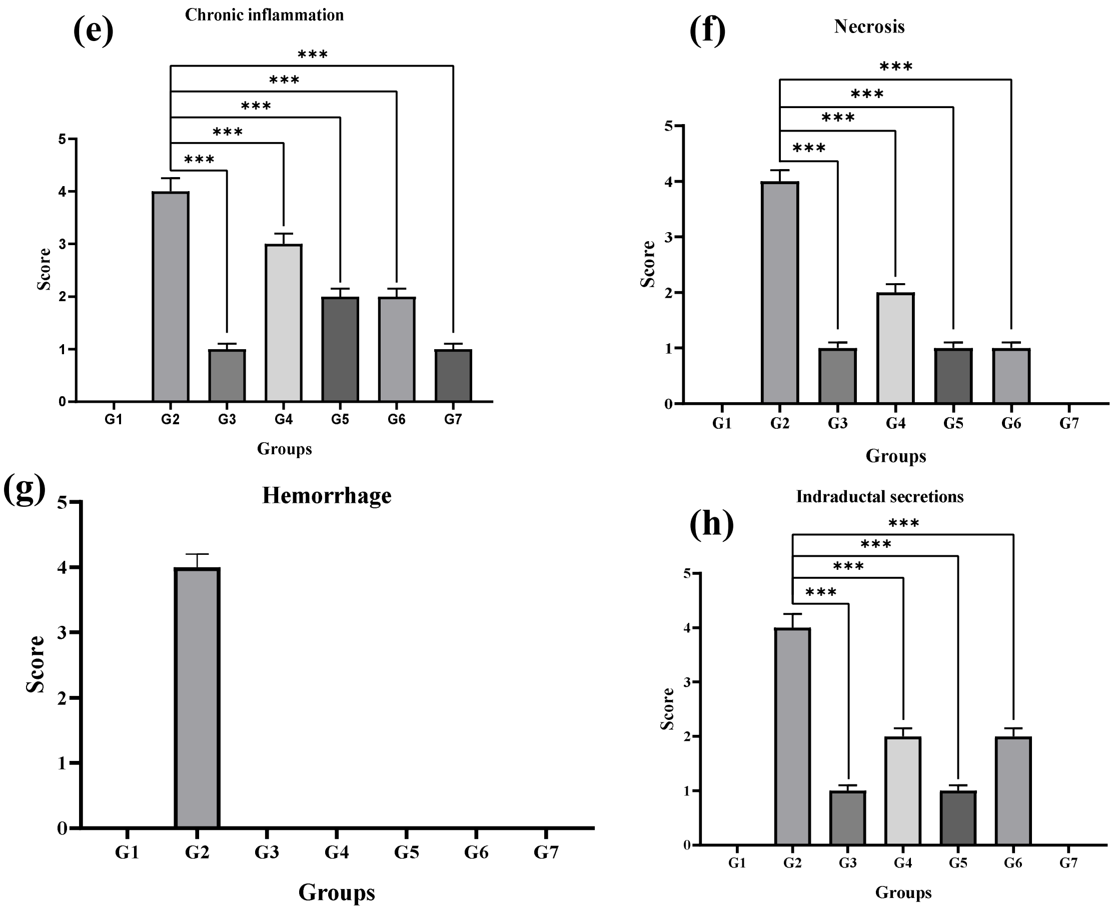

Figure S12. Histological features of different groups of breast tissues with statistical significance.

Table S1. The characteristics of the best selected phytocompounds

**Sr. No.**	**Phytochemical**	**MF**	**OB > 30%**	**DL >0.18**	**MW (g/mol)**	**PubChem ID**
1	Cycloeucalenol	$C_{30}H_{50}O$	39.73	0.79	426.7	101690
2	Cholesterol	$C_{27}H_{46}O$	37.87	0.68	386.7	5997
3	Obtusifoliol	$C_{30}H_{52}O$	42.55	0.76	426.7	65252
4	Lophenol	$C_{30}H_{50}O$	38.13	0.71	400.7	160482
5	α-spinasterol	$C_{29}H_{48}O$	42.98	0.76	412.7	5281331
6	Cycloartenol	$C_{30}H_{50}O$	38.69	0.78	426.7	92110
7	α1-sitosterol	$C_{29}H_{50}O$	43.28	0.78	426.7	9548595
8	Taraxerol	$C_{30}H_{52}O$	38.4	0.77	426.7	92097
9	Campesterol	$C_{28}H_{48}O$	37.58	0.71	400.7	173183
10	24-methylenelophenol	$C_{19}H_{48}O$	44.19	0.75	412.7	5283640
11	β-sitosterol	$C_{29}H_{50}O$	36.91	0.75	414.7	222284
12	Stigmasterol	$C_{29}H_{48}O$	43.83	0.76	412.7	5280794
13	Folic acid	$C_{19}H_{19}N_7O_6$	68.96	0.71	441.4	135398658
14	Betulinic acid	$C_{30}H_{48}O_3$	55.38	0.78	456.7	64971

MF: Molecular formula; OB: Oral Bioavailability; DL: Drug-likeness; MW: Molecular weight

Table S2. Indication of each color interaction in the PPI network of STRING.

**Interaction type**	**Color representation**	**Indication**
Known interactions		From curated database
	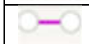	Experimentally determined
Predicted interactions		Gene neighbourhood
		Gene fusion
		Gene co-occurrence
Others	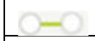	Textming
		C0-expression
	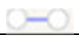	Protein homology

Table S3. Drug-like parameters or ADMET profiling of the best selected phytochemicals

**Parameters**	**Phytochemicals**		
	**Folic acid**	**Betulinic acid**	**Stigmasterol**
Absorption			
BBB	+	−	+
HIA	+	+	+
Caco-2 permeability	−	−	+
PGS	+	−	+
PGI	−	−	−
Metabolism			
CYP3A4 Substrate	+	+	+
CYP2C9 Substrate	−	−	−
CYP2D6 Substrate	−	−	−
CYP3A4 Inhibitor	−	−	−
CYP2C9 Inhibitor	−	−	−
CYP2C19 Inhibitor	−	−	−
CYP2D6 Inhibitor	−	−	−
CYP1A2 Inhibitor	−	−	−
Toxicity			
AMES Toxic	−	−	−
Carcinogenic	−	−	−
